# A soft and ultrasensitive force sensing diaphragm for probing cardiac organoids instantaneously and wirelessly

**DOI:** 10.1038/s41467-022-34860-y

**Published:** 2022-11-25

**Authors:** Quanxia Lyu, Shu Gong, Jarmon G. Lees, Jialiang Yin, Lim Wei Yap, Anne M. Kong, Qianqian Shi, Runfang Fu, Qiang Zhu, Ash Dyer, Jennifer M. Dyson, Shiang Y. Lim, Wenlong Cheng

**Affiliations:** 1 Department of Chemical & Biological Engineering, Monash University, Clayton, VIC 3800 Australia; 2 O’Brien Institute Department, St. Vincent’s Institute of Medical Research, Fitzroy, VIC Australia; 3 Department of Medicine and Surgery, University of Melbourne, Melbourne, VIC Australia; 4 The Melbourne Centre for Nanofabrication, Clayton, VIC 3800 Australia; 5 Department of Biochemistry & Molecular Biology, Biomedicine Discovery Institute, Clayton, VIC 3800 Australia; 6 Faculty of Engineering, Monash Institute of Medical Engineering (MIME), Monash University, Clayton, VIC 3800 Australia; 7 Drug Discovery Biology, Faculty of Pharmacy and Pharmaceutical Sciences, Monash University, Parkville, VIC, Australia; 8 National Heart Research Institute Singapore, National Heart Centre, Singapore, Singapore

**Keywords:** Biotechnology, Nanoscale devices, Biomedical engineering, Induced pluripotent stem cells, Characterization and analytical techniques

## Abstract

Time-lapse mechanical properties of stem cell derived cardiac organoids are important biological cues for understanding contraction dynamics of human heart tissues, cardiovascular functions and diseases. However, it remains difficult to directly, instantaneously and accurately characterize such mechanical properties in real-time and in situ because cardiac organoids are topologically complex, three-dimensional soft tissues suspended in biological media, which creates a mismatch in mechanics and topology with state-of-the-art force sensors that are typically rigid, planar and bulky. Here, we present a soft resistive force-sensing diaphragm based on ultrasensitive resistive nanocracked platinum film, which can be integrated into an all-soft culture well via an oxygen plasma-enabled bonding process. We show that a reliable organoid-diaphragm contact can be established by an ‘Atomic Force Microscope-like’ engaging process. This allows for instantaneous detection of the organoids’ minute contractile forces and beating patterns during electrical stimulation, resuscitation, drug dosing, tissue culture, and disease modelling.

## Abstract (web summary)

It is challenging to directly characterize mechanical properties of soft 3D cardiac organoids with current sensors. Here the authors report an electronic skin-based all-soft organoid-sensing system which can wirelessly monitor minute force profiles of cardiac organoids in real-time in-situ.

## Introduction

Dynamic mechanical properties of stem cell-derived cardiac organoids can provide important biological cues for understanding how the human heart tissue grows and responds dynamically to electrical stimulation (ES) and pharmacological agents. Therefore, cardiac organoids may become the next-generation model for better understanding of cardiovascular function and disease guiding development of personalized medicine^1–3^. However, it has been challenging to directly and instantaneously measure their mechanical properties in real-time and in-situ under their natural physiological microenvironments.

Optical imaging is currently the dominant method to track beatings of cardiac tissues^4–6^ either by recording their twitch motions^7,8^ or by mapping Ca^2+^ flux after staining with fluorescence dyes^9,10^ but it doesn’t measure mechanical properties directly. While mechanical models based on classic beam theories can be developed to convert videos into motions and then into forces^11–13^, super-large amount of optical imaging data storage and post-processing requirements prevent direct and real-time characterization of cardiac organoid mechanical properties.

On the other hand, technologies are being developed to integrate cardiac tissues with soft electronic sensors or so-called electronic skins. The fundamental limitation for accurate and instantaneous probing mechanical properties lies in the mismatch in mechanics and topology between soft, curvilinear tissues and rigid, planar and bulky electronic sensors. To tackle this, elastic cantilever strain sensors have been designed to detect cardiac tissue contractility^14,15^, but this method is limited to two-dimensional (2D) adherent cardiomyocytes and is not plausibly extendable to three-dimensional (3D) suspended cardiac spheroids/organoids. In recent years, there are booming advancements of cardiac bioelectronics for electrophysiological signal recording. For example, mesh-based soft sensors have been well integrated with cardiac tissues in 2D^16^ or 3D^17^; self-rolling electronics may be used to “wrap” cardiac spheroids/microtissues^18,19^, and free-standing electronics may be integrated with cardiac patches^20^. However, few studies have accomplished direct mechanical motion measurements especially for 3D cardiac spheroids/organoids. Recently, polyimide-based compliant 3D frameworks instrumented with strain sensors have been developed to measure contractility of C2C12 muscle tissues. However, the muscle tissues can only contract when being paced by electrical stimulation^21^, which are different from cardiac tissues that spontaneously beat all the time. It is non-trivial to monitor the beating forces of cardiac organoids continuously and non-invasively with currently available technologies. In another recent paper, shape-matching 3D mesostructured origami/kirigami electronics can envelop and mechanically constrain cerebral organoids, enabling improved nanoindentation-based mechanical measurement^22^. Possible perturbation of gene expression within an unnatural mechanically constrained envelope and the topological complexity of various organoids may pose a challenge to model native tissue physiological conditions. Other technologies such as fibronectin lattice mesh biosensors^23^ as well as Polydimethylsiloxane (PDMS) pillar integrated magnetic sensors^24^ could monitor contractile strain or force of cardiac tissue but require optical imaging to derive such information. Commercial instruments such as FLEXcyte 96^25,26^ system could measure the cardiomyocyte contractility through no-contact displacement induced capacitive changes but not for cardiac organoids.

Herein, we report an all-soft and ultrasensitive organoid force-sensing system. The system consists of elastic force-sensing diaphragm based on nanocracked platinum film, which is integrateable into a soft cell culture well and able to establish reliable soft contact with cardiac organoids of any ages and arbitrary sizes/shapes, in an AFM-like soft engaging process. This design concept was inspired by the human finger-based detection of artery pulse (Supplementary Fig. 1), in which a right positioning and intimate contact are crucial. While theoretically the beating forces might be detected with the previous cantilever-based sensor concept for 2D cardiomyocyte system^14,15^, the challenge to establish reliable contact between rigid 2D sensor surface with topologically complex 3D surface may limit sensitivity and reliability of the beating forces to be detected. Wireless cantilever sensors have not been reported to the best of our knowledge. Our soft-sensing system is capable of direct, reliable, sensitive and instantaneous probing of cardiac organoids’ contracting/relaxing patterns in natural cell culture well with minimal perturbation. Another attribute of our resistive sensor is its ease to integrate with wireless communication circuitry, for which we demonstrate remote monitoring of cardiac organoid beating under ES and drug dosing simply via a smartphone. We believe our soft bio-force sensing system is general, potentially extendable for detecting dynamic bio-forces from other tiny soft creatures such as lung, muscle organoids and water bears under natural bioenvironments.

## Results and discussion

### All soft organoid biosensory system

**Fig. 1:**
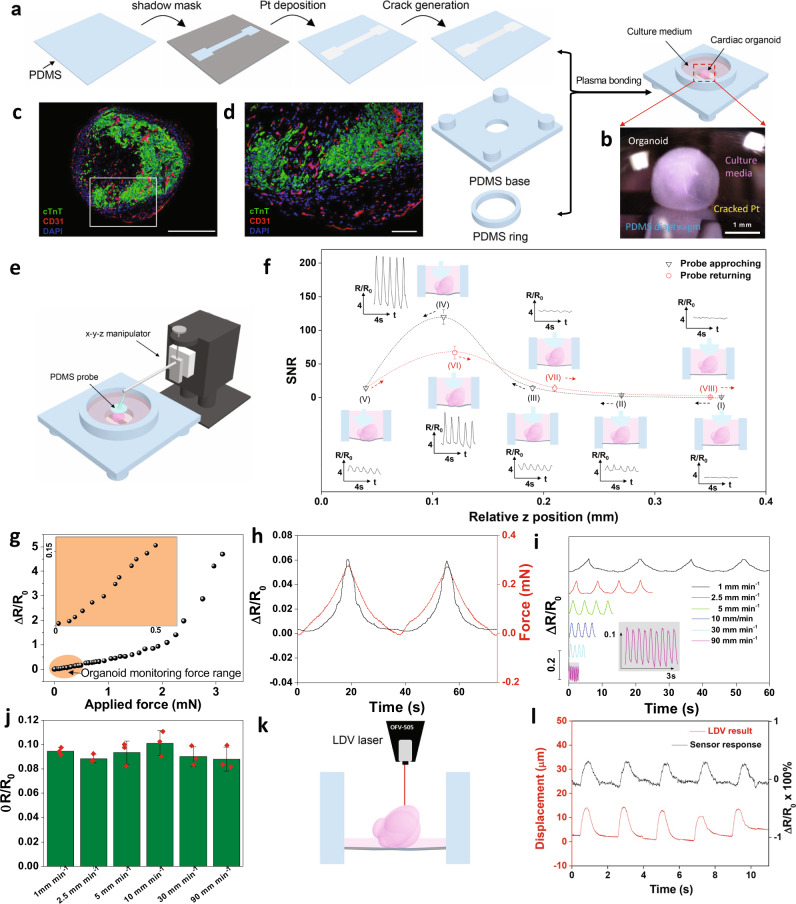
Fabrication and characterization of the elastic nanocracked force-sensing diaphragm. **a** Schematic of the fabrication process of the Pt-based elastic nanocracked force-sensing diaphragm assembled in a PDMS-based organoid culture chamber. **b** optical image of an organoid and the soft force sensing diaphragm interface. **c**, **d** Representative cross-sectional images of an engineered vascularized human cardiac organoid stained with cardiac troponin T (green, cardiomyocytes), CD31 (red, endothelial cells) and DAPI (blue, nuclei) from three independent experiments with similar results. Scale bar in b and c are 500 μm and 100 μm, respectively. **e** Schematic of the experiment set-up of instantaneous organoid beating monitoring by the Pt-based force-sensing diaphragm with soft PDMS probe and *x*–*y*–z manipulator. **f** ‘AFM-like’ engaging process to achieve high SNR when the probe was approaching the organoid (position (I–V)) and departing the organoid (position (V–VIII)) and the representative resistance changes of the force-sensing diaphragm align with different *z*-position. **g** Relationship between applied force and sensor resistance ratio under ‘optimal contact’ condition. **h** Dynamic force changes with sensor resistance changes under ‘optimal contact’ condition. **i** Sensor resistance changes in response to a force of 0.3 mN with different loading-unloading velocity from 1 mm min^−1^ to 90 mm min^−1^. **j** Comparison of the amplitude averaged from 30 waveforms with different loading-unloading velocity. Data are presented as mean ± SEM (*n* = 3). **k** Schematic of the experiment set-up for instantaneous organoid beating measurement simultaneously by LDV and the force-sensing diaphragm. **l** Comparison of the displacement changes from LDV and resistance changes from the force-sensing diaphragm.

Unlike 2D cardiomyocytes, it is more challenging to directly measure contracting/relaxing mechanical properties of 3D cardiac organoids, given their miniaturized sizes, uneven surfaces in 3D, free-floating nature in culture media, and dynamic changes in volume and morphology during development. To tackle this, we have designed an all-soft cell culture well with an ultrasensitive resistive sensing diaphragm integrated (Fig. 1a). PDMS is chosen as a soft and stretchable substrate to minimize possible damage of fragile cardiac organoids. Nanocracked Pt film is used as resistive sensing elements due to its simplicity in fabrication, strong adhesion to PDMS and superior sensitivity. Such designed Pt sensor can be patterned via shadow mask lithography and be easily integrated with other PDMS components to obtain an all-PDMS cell culture well via an oxygen plasma-assisted bonding process (Fig. 1a and Supplementary Fig. 2). Millimeter-sized cardiac organoids can be transferred into this soft cell culture well instrumented with the force sensing diaphragm (Fig. 1b–d).

In order to characterize organoids that are fully immersed in cell culture media, we devised an AFM-like engagement process with a soft PDMS probe controlled by an *x*–*y*–*z* positioner (Fig. 1e). This offers a simple yet efficient strategy to establish conformal soft sensing diaphragm-organoid microcontact—crucial for the reliable detection of cardiac organoid beating (Supplementary Video 1). Once conformal soft ‘sandwich’ contact was established, subtle changes in cardiac organoid beating behaviors associated with contraction and relaxation could be precisely and reliably detected by the highly sensitive nanocracked Pt diaphragm, which is based on the electrical resistance changes induced by cardiac contraction and relaxation events (Supplementary Fig. 3)^27^. In operation, a dimensionless signal-to-noise ratio (SNR) value was used to gauge the degree of organoid-diaphragm contact (see Supplementary Fig. 4 for SNR definition). In the trace cycle (black dashed line in Fig. 1f), as the upper PDMS probe approached the cardiac organoid, the SNR value experienced a gradual increase (I → II → III) and sudden increase (IV), followed by a decrease (V). This reflected the process of “no contact” (I), “just contact” (II), “minor contact” (III), “optimal contact” (IV) and “over contact” (V) between the organoid and sensing diaphragm. In this experiment, the best soft conformal microcontact was established with the highest SNR of >130 and the base electrical resistance had a shift of ~8% (Supplementary Fig. 5). This optimal soft microcontact force was estimated to be ~0.67 mN (Supplementary Figs. 5, 6). The overload could impede the contractility of the cardiac organoid, leading to a reduced SNR (position V in Fig. 1f). Interestingly, the soft engagement process could be reversed in the retrace cycle by gradually reducing the load (Fig. 1f, red dashed line V → VI → VII → VIII).

To further prove the sensitivity and fidelity of our culture well-integrated soft diaphragm sensors, we applied an external force over a non-beating organoid placed on top of the diaphragm under “optimal contact” conditions and simultaneously recorded sensor responses (Fig. 1g and Supplementary Fig. 7). It is known that contractile forces of cardiac organoids are typically 0.15–0.5 mN^11,12,28^, in which our sensing diaphragm exhibited an excellent linear relationship with the applied pressure at a high sensitivity of 5.64 kPa^−1^ and a high gauge factor of 338.9 (Section I–4 and Supplementary Figs. 7, 8). The high sensitivity originates from the stress-induced nano-crack propagation (Supplementary Fig. 9) of the nanocracked Pt thin film. In addition, the relative resistance change *(R* *−* *R*_*0*_*)/R*_*0*_, where *R* is the electrical resistance of the film and *R*_*0*_ is the resistance (when no force or pressure is applied) of the force-sensing diaphragm aligns well with the applied force of 0–0.3 mN (Fig. 1h), demonstrating negligible hysteresis. In addition, our diaphragm also exhibited good linearity with the applied pressure at the “no contact” condition, with slightly decreased force sensitivity compared with that at the “optimal contact” condition (Supplementary Fig. 10).

A typical cardiac organoid has a beating frequency of 0.5–3 Hz, for which we validated the reliability and fast responses of our resistive force-sensing diaphragm by applied dynamic strains with various frequencies. In the loading-unloading cycles with strains applied with various velocities of 1–90 mm/min (equivalent to a constant frequency range of 0.06–3 Hz), the relative resistance response is stable and independent of the loading frequencies (Fig. 1i, j). The sensing diaphragm was also very durable, showing stable responses before and after 1000 repeated loading-unloading cycling tests (Supplementary Fig. 11).

While the sensing diaphragm has adequate sensitivity and fidelity for detecting contractile forces of cardiac organoids, reliable contact between sensing diaphragm and organoids is the prerequisite. Unfortunately, unlike 2D cardiomyocytes, our 3D cardiac organoids are cultured in suspension in medium with minimal contact with the bottom of the cell culture well. By reducing the volume of culture medium, improved organoid-diaphragm contact could be established via water surface tension. This allowed simultaneous measurement of contractile displacements by Laser Doppler Velocimetry (LDV) and resistive signals from the sensing diaphragm (Fig. 1k). Their matching line profiles further demonstrated the reliability of our force-sensing diaphragm in real-time detection of cardiac organoid beating events (Fig. 1l). Under the optimal soft microcontact, the cardiac organoid could be monitored in their native condition in the CO_2_ incubator continuously for at least 7 h (Supplementary Fig. 12).

### Validation and simultaneous multi-modal measurement

Cardiac organoids used in this study exhibited synchronous beating, which was confirmed by optically imaging the organoid suspended in culture medium from its top, bottom and side views (Supplementary Fig. 13). Through the video analysis, we proved that the beating frequencies were consistent and independent from the viewing angles of the cardiac organoids (Supplementary Fig. 14 and Supplementary Video 2). Such synchronous beating organoids rendered it possible for us to combine our diaphragm sensors with other analytical platforms such as optical imaging and microelectrode array (MEA) for multi-modal analysis of the cardiac organoids.

**Fig. 2:**
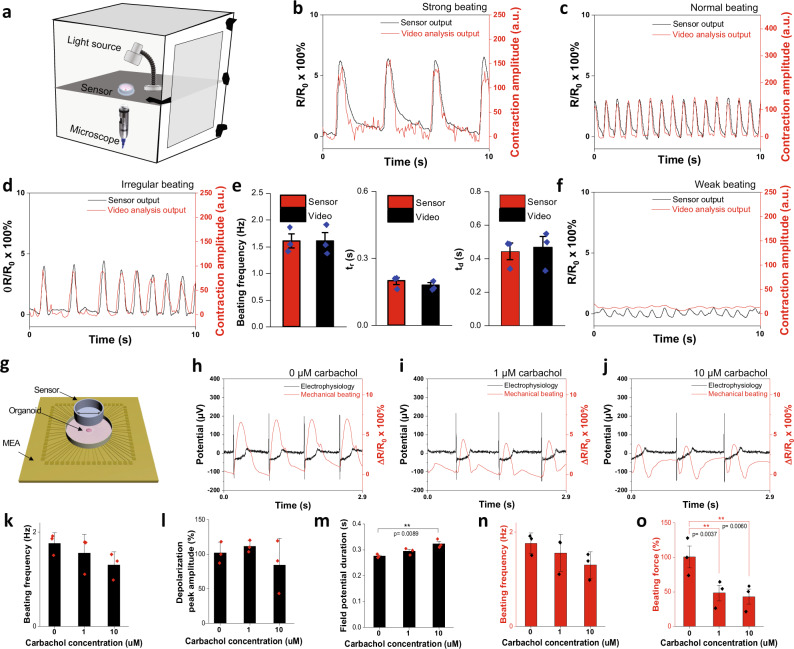
Simultaneous optical/force measurements and electrophysiological/force measurements. **a** Schematic of the simultaneous optical/force measurements set-up. **b**–**d** Simultaneous optical/force measurements with strong beating (**b**), normal beating (**c**), and irregular beating (**d**), **e** Changes in beating frequency (left), rise time (t_r_, middle), and decay time (t_d_, right) of the sensor and video output owing to the contraction and relaxation of the 3-day old cardiac organoids (*n* = 3). **f** Simultaneous optical/force measurements with weak beating. **g** Schematic of the simultaneous electrophysiological/force measurements set-up. **h–j** Simultaneous electrophysiological/force measurements of an organoid before carbachol treatment (**h**), after 1 μM carbachol (**i**) and after 10 μM carbachol (**j**) treatment. **k–o** The changes in beating frequency (**k**), depolarization peak amplitude (**l**) and field potential duration (**m**) from electrophysiological measurements, as well as beating frequency (**n**) and relative beating force (**o**) from sensor measurements (*n* = 3). Data are presented as mean ± SEM. Statistical analysis was performed using one-way ANOVA (*p < 0.05, **p < 0.01, ***p < 0.001).

We have designed a proof-of-concept system capable of simultaneous optical and electrical measurements inside a conventional incubator (Fig. 2a). In this system, a digital microscope was used to record organoid beating motions and produce standard beating patterns, similar to conventional microscopes. Our diaphragm sensors could simultaneously record mechanical forces associated with cardiac organoid contraction. Remarkably, the electrical output from the diaphragm sensor was well aligned with the optical measurement results in organoids exhibiting different beating force and regularity (Fig. 2b–d and Supplementary Video 3, 4). Other beating parameters, such as beating frequency, rise time (*t*_*r*_), decay time (*t*_*d*_), were also well aligned between sensor and simultaneous video recording outputs (Fig. 2e). These results validate the reliability and accuracy of our sensors.

Further measurement on weakly beating organoids demonstrated higher sensitivity of the diaphragm sensors than the standard optical recording (Fig. 2f and Supplementary Video 5). The contraction profile of a weakly beating organoid could not be detected by the optical imaging process (red line, Fig. 2f) but was clearly identified by our diaphragm sensor (black line, Fig. 2f). In a separate experiment, an abnormal contraction profile could be detected in cardiac organoids exhibiting dyssynchronous contraction by our sensor but not by the optical imaging (Supplementary Fig. 15). The double peak contraction profile was likely attributed to two beating foci in contact with the sensor, which cannot be detected by optical imaging process that analyze the pixel displacement at the edge of the organoid^29^. This observation further highlighted the sensitivity and attributes of our sensor over conventional optical video imaging (Supplementary Table S1).

In addition, a lactate dehydrogenase (LDH) assay was carried out to determine if mechanical pressing forces applied on the cardiac organoid caused tissue damage. The LDH levels were comparable in organoids cultured for 24 h in the PDMS chamber, compressed by the PDMS probe, or cultured in a conventional polystyrene (PS) culture plate without applying a PDMS probe (Supplementary Fig. 16), indicating negligible tissue damage from the PDMS culture well or mechanical compression.

Our diaphragm-based sensing concept is versatile and could be also positioned on the top of the cardiac organoid for detecting its contractile activities. This offered the unprecedented opportunity for simultaneous electrophysiological and force measurement by coupling our top-positioned diaphragm sensor with a commercial MEA device (Fig. 2g). Using carbachol as a chronotropic and inotropic agent, we correlated the electrophysiological and mechanical signals before and after drug administration. The time-lapsed resistive patterns associated with our diaphragm sensors were reliable and correlated well with MEA potential profiles, revealing the relationship between mechanical and electrical properties of the cardiac organoids (Fig. 2h–j).

Both MEA and our diaphragm sensors detected negative chronotropic and inotropic effects of carbachol. Treatment with 10 μM of carbachol reduced the beating frequency (Fig. 2k, n), force (Fig. 2o) and elongated the field potential duration (Fig. 2m) of the cardiac organoids without significantly altering the amplitude of the depolarization peak of organoid extracellular field potential (Fig. 2l). These chronotropic effects of carbachol on beating frequency and field potential duration are consistent with what is observed in the literature^30^.

### Real-time monitoring effect of electrical stimulation on cardiac contractility

**Fig. 3:**
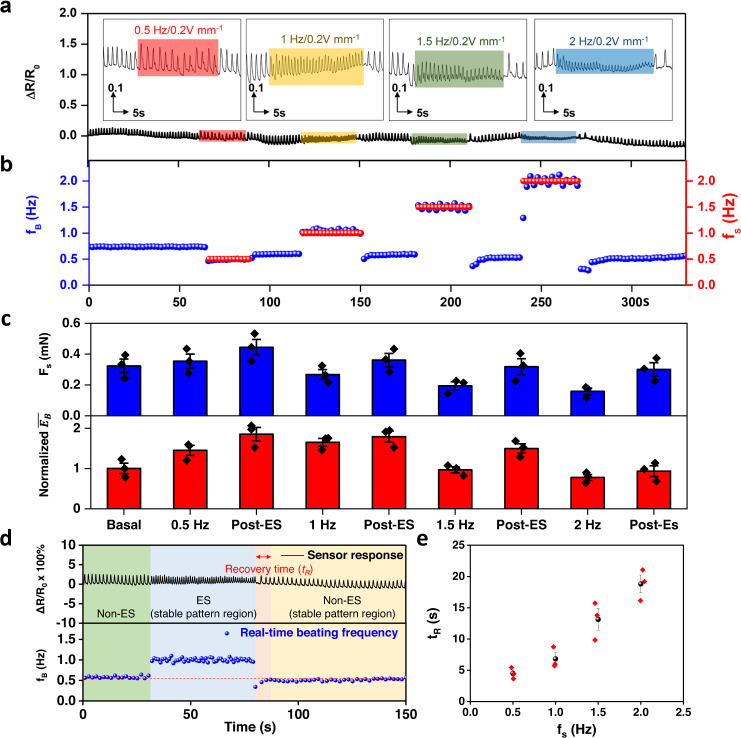
Instantaneous measurement of cardiac organoids during electrical stimulation in ambient conditions. **a** Real-time resistance changes of the sensor during periods of ES with different frequencies. left to right: 0.5 Hz, 1 Hz, 1.5 Hz, and 2 Hz with an electrical field strength of 0.2 V mm^−1^; **b** Real time beating frequency changes of the organoid (blue dot) during electrical stimulation (red dot). **c** The calculated contractile force (*F*_*s*_) and normalized energy consumed from straining events per second ($\bar{{E}_{B}}$) of each state for a particular organoid. (*n* = 3) **d** An example of the current response of the soft sensor (top) and the corresponding real-time beating frequency (*f*_*B*_) of a cardiac organoid during ES process. **e** Comparison of the recovery time after each ES was removed (*n* = 3). Data are presented as mean ± SEM.

Our diaphragm-based sensing system was capable of identifying fine details of dynamic beating patterns of engineered vascularized cardiac organoids instantaneously in responses to changes in ES stimulation frequency (*f*_S_) and duration (*t*_S_) and electrical field strength (*V*_S_). The time-lapse ES frequency-dependent pattern demonstrated the performance of our sensing system in instantaneous detection of cardiac organoid beating profiles (Fig. 3a). It shows that the cardiac organoid beating frequency (*f*_B_) was almost synchronized to the ES frequencies (*f*_B_ = *f*_S_) (Fig. 3b). A typical electrical profile recorded by the sensor had a Gaussian peak. By assuming the uniform contraction/relaxation process, we derived a ‘balloon’ model correlating half of the Gaussian peak area with the straining work imposed on diaphragm sensors by the cardiac organoids (Supplementary Section III-1 and Supplementary Figs. 17, 18). Hence, the peak area of the *I*–*t* curve recorded could be used to describe the energy consumption ($\bar{{E}_{B}}$) associated with organoid beating events. Based on the linear relationship between the applied force and current changes of the diaphragm sensor in the cardiac organoid beating force range (Fig. 1g), we also estimated the contractile force (*F*_*s*_) to be proportional to the beating amplitude (*A*_B_) (Supplementary Section III-2). Both $\bar{{E}_{B}}$ and *F*_*s*_ reduced with an increased stimulation frequency at supra-physiological pacing rates (between 1 and 2 Hz) (Fig. 3c), indicating a negative force-frequency response. In turn, it suggests a slowed calcium handling in the immature cardiomyocytes derived from pluripotent stem cells and that supra-physiological pacing may trigger a pathological response in these immature cardiomyocytes^11,28,31,32^. However, upon ES removal, both $\bar{{E}_{B}}$ and *F*_*s*_ recovered to approximately their basal levels albeit with a small fluctuation, which indicated recovery of the cardiac organoid’s intrinsic electromechanical function.

Importantly, subtle details after ES removal could be identified by our sensors, which is difficult to achieve with other conventional methods such as optical video recording (Fig. 3d). It shows that a stable regular beating pattern (non-ES region) rapidly adapted to a fresh pattern when subjected to ES paced to ES frequency (ES region), which became unstable upon ES removal (recovery) for a certain duration (*t*_R_) before the stable pattern established (Post-ES). *t*_R_ is defined as the time difference between cessation of ES and recovery of basal beating frequency before ES. *t*_R_ was increased from ~4.5 s to ~18.8 s when *f*_S_ increased from 0.5 to 2 Hz (Fig. 3e), suggesting a longer duration is needed for the cardiac organoids to restore their basal beating frequency following a higher pacing frequency.

With our instantaneous soft biosensing system, fine mechanical properties of organoid beating in response to ES duration (*t*_S_) (Supplementary Fig. 19) and ES field strength (Supplementary Fig. 20) could also be characterized in real-time. It shows that synchronization of cardiac organoid beating frequency with the stimulation frequency is independent of ES duration. However, the beating frequency could not be paced by ES if the applied field potential was too low at 0.1 V mm^−1^ (Supplementary Fig. 20b). Nevertheless, a post-ES recovery time was still needed for the cardiac organoids to restore their basal beating frequency, which became much longer after a threshold ES duration (~90 s for the particular organoid tested) (Supplementary Fig. 19b). It appeared that the electromechanical function also improved above the threshold ES duration (Supplementary Fig. 19c).

### Real-time monitoring of organoid beating restoration

The instantaneous sensing capability of our system was further demonstrated through detection of the dynamic contracting/relaxing pattern during an electrical resuscitation process for cardiac organoids that lost their spontaneous beating activity after prolonged culture (Supplementary Fig. 21). While the non-beating cardiac organoid could be paced at *f*_S_ = 1 Hz, no spontaneous beating activity could be established upon ES removal even when the ES duration increased to 120 s (Supplementary Fig. 21a, b). Interestingly, after ES at 2 Hz for 60 s, the spontaneous beating activity was successfully resuscitated. The close-up view shows fine details of the restored cardiac organoid beating patterns, indicating a slow, irregular beating frequency upon removal of ES, which gradually established a regular beating pattern after about 71 s. This electrically resuscitated cardiac organoid displayed a similar response to ES field strength, frequency, and duration as normal healthy cardiac organoids (Supplementary Figs. 21c and 22–24). Nevertheless, there were some subtle differences that could be identified with our soft sensing system. For example, the recovery time for the resuscitated cardiac organoid appeared to be longer than the healthy one (Supplementary Fig. 21c). In addition, the beating rate variability of the resuscitated cardiac organoid was higher than the healthy one both in the non-ES and ES conditions (Supplementary Fig. 25).

### Long-term organoids monitoring with drug dosing

**Fig. 4:**
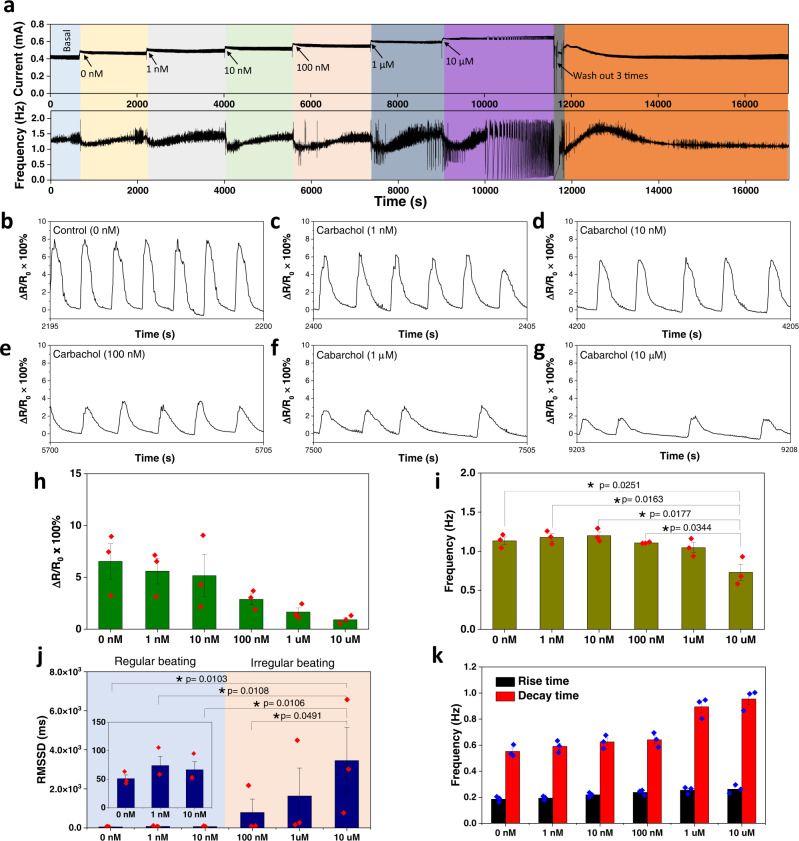
Instantaneous measurement of cardiac organoids treated with a chronotropic agent. **a** Continuous real-time readout of the beating events and the corresponding beating frequency changes from a cardiac organoid treated with cumulative doses of carbachol from 1 nM to 10 μM. **b–g** Enlarged views of the changing beating patterns 3 min after 0 nM (**b**), 10 nM (**c**), 100 nM (**d**), 1 μM (**e**), and 10 μM (**f**) carbachol dosage in a time-dependent manner. **h**–**k** Bar plot depicting the resistance changes (**h**), frequency changes (**i**), RMSSD (**j**), and rise time/decay time (**k**) of the organoids (*n* = 3). Data are presented as mean ± SEM. Statistical analysis was performed using one-way ANOVA with a Bonferroni post hoc test (*p < 0.05, **p < 0.01, ***p < 0.001).

We further extended our conformal soft sensing platform to examine the responsiveness of our engineered cardiac organoid to pharmacological treatment with and without concomitant ES in real-time. Firstly, chronotropic and inotropic responses to increasing doses of carbachol were evaluated in a cardiac organoid on day 9–10. The dynamic changes in beating patterns and beating frequency over a duration of 17,000 s were recorded continuously (Fig. 4a), displaying a reduction in beating frequency and relative contraction force (Fig. 4b–i) generated by the cardiac organoid in a dose-dependent manner. After addition of carbachol, the beating frequency of organoids decreased rapidly until reaching a maximum effect of carbachol before gradually return to the basal level. An arrhythmic beating pattern was observed when the cardiac organoid was treated with high concentrations of carbachol (Fig. 4f, g and Supplementary Figs. 26–31). At 100 nM carbachol, the time-lapse recording showed an irregular beating pattern starting from ~5 min after drug administration and became more apparent after 10–20 min (Supplementary Figs. 26, 27), which is also confirmed by the increase of heart rate variability parameter RMSSD, which was calculated over a period of 20 min after addition of carbachol (Fig. 4j). These pro-arrhythmic, negative chronotropic and inotropic responses to carbachol were more pronounced when cardiac organoids were treated with higher concentrations of carbachol at 1 and 10 µM (Fig. 4j), and this is in agreement with previous studies^33–35^. Furthermore, the increasing dose of carbachol yielded a substantial increase in the decay time of the organoid beating, indicative of the decrease in force generation at higher carbachol concentrations (Fig. 4k).

Next, our soft sensor was applied to characterize whether ES could restore the regular beating rhythm of the carbachol-treated cardiac organoid, which developed arrhythmia at high concentration of carbachol. Our results of real-time beating patterns show restoration of rhythmic contraction profile after being subjected to ES pacing at all frequencies tested, especially when *f*_S_ > *f*_B_, but arrhythmia resumed upon removal of ES pacing (Supplementary Fig. 32a, b). Close scrutiny of the beating patterns revealed that the recovery time *t*_R_ increased from ~3 s at *f*_S_ = 1.5 Hz to 20 s at *f*_S_ = 3 Hz (Supplementary Fig. 32c). It might suggest higher stimulation frequency induced a higher degree of cellular stress in carbachol-treated cardiac organoids. Further scrutiny of “stable pattern regions” during ES and post-ES showed that the relative contraction force decreased with increasing *f*_S_ (Supplementary Fig. 32d). This negative force-frequency response is consistent with that from above-investigated healthy organoids.

Our soft microcontact sensing platform is also capable of monitoring changes in contraction profile of cardiac organoids in real-time in the presence of mechanical disturbances such as during the exchange of cell culture media (Supplementary Fig. 33a). Although medium exchange caused a significant background current spike (Supplementary Fig. 33b), contractile patterns can still be detected reliably as demonstrated by the negative chronotropic effect of carbachol (Supplementary Fig. 33c), which is consistent with the aforementioned results by the administration of carbachol in a gentle dropwise manner.

### Disease modeling

**Fig. 5:**
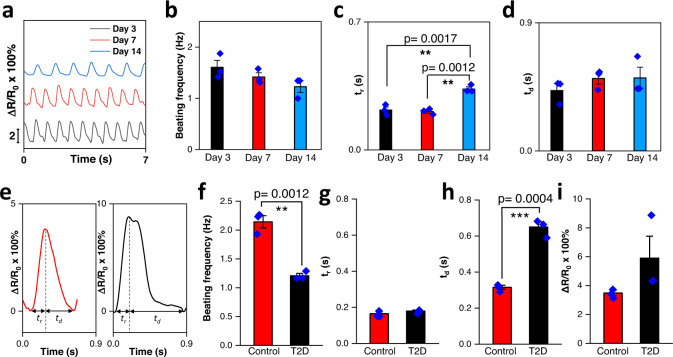
Developmental studies during tissue culture and diabetic disease modeling. **a** Representative contraction profiles showing changes in resistance ratio of the sensor induced by the contraction and relaxation of an organoid at day 3, 7, and 14. **b**–**d** Beating frequency (**b**), rise time (**c**), and decay time (**d**) of organoids at day 3, 7, and 14 obtained from sensor output (*n* = 3). **e** Representative waveform patterns of organoids in control group (left) and diabetic group (right) at day 3. **f**–**i** Beating frequency (**f**), rise time (**g**), decay time (**h**) and beating force (**i**) of organoids in control and diabetic (T2D) groups obtained from sensor output (*n* = 3). Data are presented as mean ± SEM. Statistical analysis was performed using one-way ANOVA with a Bonferroni post hoc test or Student’s *t*-test (*p < 0.05, **p < 0.01, ***p < 0.001).

We extended our soft sensing platform to evaluate the contractile function of organoids over 14 days in culture. The beating frequency of the organoid decreased as it aged (Fig. 5a, b), indicative of increased maturity of cultured organoids. Simultaneously, both the rise time (*t*_*r*_) and decay time (*t*_*d*_) gradually increased and reached maximum value on day 14 (Fig. 5c, d).

We further applied our diaphragm sensors to evaluate the beating profiles of organoids subjected to type 2 diabetic (T2D) conditions to highlight its potential disease modeling applications. Organoids were randomized to either control, or T2D conditions consisting of 20 mM glucose and pathological concentrations of fatty acids (see methods for details). Figure 5e shows the real-time traces of contraction and relaxation characteristics of organoids in the control group (red line) and in the T2D group (black line) measured using the diaphragm sensor 72 h post diabetic conditions treatment. As shown in Fig. 5f, the beating frequency of the T2D group is significantly lower than that of the control group. Cardiac organoid rise time was unchanged by T2D conditions compared to control (Fig. 5g), however, decay time was significantly lengthened (Fig. 5h). These data are consistent with the clinical presentation of diabetic cardiomyopathy, in which diastolic function (decay time), but not systolic function (rise time), is impaired in the early stages of the disease^36^. Beating force was unchanged due to T2D conditions (Fig. 5i). These data confirm that our diaphragm sensor is able to detect clinically relevant pathological changes in organoid contraction/relaxation events.

### Remote organoid monitoring wirelessly

**Fig. 6:**
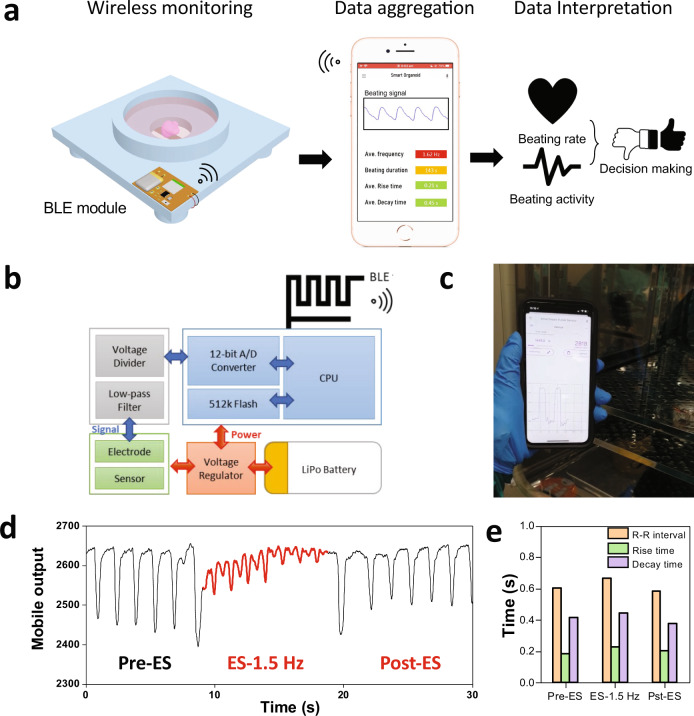
Integration of wireless BLE module to the biosensory system. **a** Full spectrum-procedures of organoid biosensory system integrated with wireless BLE module for remote organoid monitoring and decision making. **b** Block diagram of the smart-organoid monitoring system, including the nRF52832 Bluetooth Low Energy SoC for wireless data transmission to a smartphone, a lithium polymer battery to power the system, and a mobile phone with Smart Organoid Monitoring software. **c** A video clip of the wireless monitoring of organoid beating events when it is cultured in the incubator. Scale bar: 2 cm. **d** Real-time output of organoid beating via mobile phone interface. **e** Bar plot depicting the *R*–*R* interval and rise time/decay time of the beating organoid pre-, during and post ES.

One attribute of resistive sensors lies at their facile integration with wireless data transmission modules, as they have low power consumption. This means the real-time organoid monitoring could be, in principle, conducted anytime and anywhere via a smartphone, offering the capability of remote organoid diagnostics for real-time decision making (Fig. 6a). As a proof of concept, we developed a flexible printed circuitry board (PCB) system containing a Bluetooth Low Energy module (BLE) and integrated it with the sensing diaphragm (Fig. 6a, b). The developed PCB consists of a lithium polymer battery to power the system and a microcontroller to process data, as well as a low-pass filter to screen data at low frequency.

With this wireless monitoring system, spontaneous beating activity of a cardiac organoid was successfully monitored in real-time remotely using a custom designed Smart Organoid Monitoring software installed in a smartphone (Fig. 6c and Supplementary Video 6). Furthermore, we also demonstrated the feasibility of wirelessly monitoring organoid beatings during ES process via the smartphone interface (Fig. 6d and Supplementary Video 7). Upon application of ES at 1.5 Hz, the paced cardiac organoid contractions matched the ES frequency accompanied with a reduced beating amplitude. Upon cessation of ES, the beating activity of cardiac organoid gradually returned to the basal spontaneous beating rate and amplitude. Furthermore, the key parameters including R–R interval, rise and decay times could be derived from the raw wireless signals (Fig. 6e). This demonstrated the facility of our wireless cardiac organoid probing system, suggesting the potential to remotely monitoring the contractile activity of cardiac organoids (Fig. 6c–e).

In summary, we report a simple yet efficient strategy to fabricate an all-soft organoid-sensing well, which is made from only PDMS and platinum film with minimal disturbance of the natural organoid-culture environment. The PDMS-supported nanocracked platinum film serves as the force-sensing diaphragm, offering superior sensitivity and fast responses that are required for instantaneous detection of cardiac organoid contraction/relaxation events. The AFM-like soft engagement enables the establishment of reliable organoid-diaphragm contact—vital for achieving a high signal-to-noise ratio. Besides, compared to previous cantilever indentation system^22,37^ to measure biomechanical properties of organoids, this AFM-like soft engagement approach to immobilize the non-adherent organoids is advantageous. It is fast, convenient, reversible and can be applied to a single organoid in a natural culture dish condition. Despite its simplicity, our sensor is powerful, able to instantaneously identify key mechanical features of cardiac organoid contraction/relaxation process (e.g., beating frequency, strength, regularity of beating patterns, etc.) under complex microenvironments including electrical stimulation, electrical resuscitation, drug dosing, tissue culture, and disease modeling. Our demonstrated capability of the wireless and remote monitoring of ‘in-culture’ organoids inside a cell culture incubator heralds a very promising future for automatic virtual tissue culture monitoring systems anytime anywhere. We believe that our soft electronic sensor design represents a potentially general route to real-time detection of small-scale biological forces. It may be extended to detect biological forces from other targets such as lung and muscle tissues, and even forces from small animals such as worms and water bears.

## Methods

### Human induced pluripotent stem cells (hiPSCs) culture and cardiomyocyte differentiation

This research project complied with all the relevant ethical regulations, and the ethics for all protocols using hiPSCs was approved by the St Vincent’s Hospital Melbourne Institutional Biosafety Committee (Exempt Dealing Registration number 21). The human iPS-Foreskin- 2 cell line, kindly provided by James A. Thomson (University of Wisconsin)^38^ was maintained on vitronectin-coated plates in TeSR-E8 media (Stem Cell Technologies) according to the manufacturer’s protocol. Cardiomyocytes were derived from human iPSCs as previously described with modifications^39^. Briefly, iPSCs were seeded onto Matrigel (Corning) coated plates at a density of 1.25 × 10^5^ cells/cm^2^ in TeSR-E8 media supplemented with 10 μM Y − 27632 (Tocris Bioscience). After 48 h when the cells were 100% confluent, which is referred to as day 0, media was replaced with RPMI 1640 basal media (Thermo Fisher Scientific) containing B-27 without insulin supplement (Thermo Fisher Scientific), growth factor reduced Matrigel (1:60 dilution) and 10 μM CHIR99021 (Cayman Chemical). At day 1, media was replaced with RPMI 1640 basal media containing B-27 without insulin supplement. At day 2, media was changed to RPMI 1640 basal media containing B-27 without insulin supplement and 5 μM IWP2 (Tocris Bioscience) for 72 h. From day 5 onwards, cells were cultured in RPMI 1640 basal media containing B-27 supplement (Thermo Fisher Scientific) and 200 μg/mL L-ascorbic acid 2-phosphate sesquimagnesium salt hydrate (Sigma-Aldrich), referred to as cardiomyocyte media, and media was changed every 2–3 days. At day 12, cardiomyocytes were dissociated into single cells and split 1:2 onto Matrigel-coated plates in DMEM/F-12 GlutaMAX media supplemented with 20% fetal bovine serum (Sigma-Aldrich), 0.1 mM 2-mercaptoethanol, 0.1 mM nonessential amino acids, 50 U/mL penicillin/streptomycin and 10 μM Y − 27632. At day 13, media was changed to cardiomyocyte media. From days 14–19, cardiomyocytes were enriched to >95% cardiac troponin T positive cells by culture in glucose-free DMEM media (Thermo Fisher Scientific) containing 4 mM lactate (Sigma-Aldrich).

### Endothelial cell differentiation from hiPSCs

Human iPSCs were differentiated into CD31 + endothelial cells^40^. For endothelial differentiation, iPSCs were dissociated into single cells and seeded onto Matrigel-coated plates at a density of 1 × 10^5^ cells/cm^2^ in TeSR-E8 media supplemented with 10 μM Y − 27632. After 24 h, referred to as day 0, media was replaced with DMEM/F12 GlutaMAX media (Thermo Fisher Scientific) containing N-2 supplement (Thermo Fisher Scientific), B27 supplement, 8 mM CHIR99021 and 25 ng/mL BMP4 (STEMCELL Technologies) for 3 days. Media was then replaced with StemPro-34 SFM complete media (Thermo Fisher Scientific) supplemented with 200 ng/mL VEGF-165 (Peprotech) and 2 µM forskolin (Sigma-Aldrich) for 3 days. At day 6, hiPSC-derived endothelial cells were dissociated into single cells, resuspended in fluorescence-activated cell sorting (FACS) buffer (0.5% (w/v) BSA, 2 mM EDTA in DPBS) and incubated with conjugated FITC Mouse Anti-Human CD31 (1:10 dilution, #555445, clone WM59, BD Pharmingen, lot#8212882) for 30 mins. Stained cells were resuspended in FACS buffer, passed through a 40 µm mesh, and sorted using a BD FACS Aria III (Supplementary Fig. 34). An unstained cell sample was used as a negative control for gating. CD31 positive cells expanded on human fibronectin (Merck) coated plates and cultured in EGM2-MV media (Lonza) supplemented with 50 ng/mL VEGF-165.

### Engineered human cardiac organoids

To construct the cardiac organoids, enriched day-19 iPSC-derived cardiomyocytes were seeded onto Matrigel-coated 48-well Nunc^TM^ UpCell plates at 2 × 10^5^ cells/cm^2^ in DMEM/F-12 GlutaMAX media supplemented with 20% fetal bovine serum (Sigma-Aldrich), 0.1 mM 2-mercaptoethanol, 0.1 mM nonessential amino acids, 50 U/mL penicillin/streptomycin and 10 μM Y − 27632. After 24 h, iPSC-derived endothelial cells (8.6 × 10^4^ cells/cm^2^) were then seeded onto the iPSC-cardiomyocytes and cultured in cardiac organoid medium consisting of a mixture of cardiomyocyte media and EGM2-MV media supplemented with 50 ng/mL VEGF-165 (at 1:1 ratio). After 24 h, the UpCell plates were brought to room temperature and the detached cell sheet was transferred to an ultralow attachment plate (Sigma-Aldrich) containing cardiac organoid media for 24 h. The resulting spheroids were then embedded in 15 µL of growth factor reduced Matrigel and cultured in cardiac organoid media. Cardiac organoids were maintained in culture in a humidified CO_2_ incubator on an orbital shaker at 100 rpm and media was changed every 2–3 days.

### Immunohistochemistry

Cardiac organoids were fixed in 4% paraformaldehyde for 1 h at room temperature and then dehydrated in 20% sucrose solution for 24 h. Dehydrated samples were embedded in Optimal Cutting temperature compound and cryosection (10 µm thick) were treated with 0.2% Triton X-100 permeabilization buffer and Ultra V block solution (Thermo Fisher Scientific) before double stained with cardiac troponin T (2 μg/mL, rabbit polyclonal, ab45932, Abcam, lot#GR3394585-1) and CD31 (2 μg/mL, mouse monoclonal, M0823, clone JC70A, DAKO, lot#00093760) followed by Alexa Fluor-488-conjugated goat-anti-rabbit (10 μg/mL, Invitrogen, lot#2247988) and Alexa Fluor-594-conjugated goat-anti-mouse (10 μg/mL, Invitrogen, lot#2043369). Sections were then counterstained with 1 μg/mL of DAPI (Invitrogen) for nuclear staining. Epifluorescence images of immunostained sections were acquired with an Olympus BX61 upright microscope using analySIS software.

### Diabetes conditions and control conditions

To induce a type 2 diabetes cardiac phenotype, day 7 cardiac organoids were randomized to either simulated diabetic culture conditions (cardiac organoid media with a final concentration of 20 mM glucose, 0.25 mM palmitate, 0.1 mM oleic acid and 0.1 mM linoleic acid) or control conditions (cardiac organoid media with 5.55 mM glucose, 14.45 mM mannitol and vehicle controls) for 72 h.

### Fabrication of the nanocracked Pt diaphragm sensor

A modified approach was used as described in the literature^27^. Firstly, a 20 µm thick PDMS layer was fabricated by spin-coating PDMS precursors (base: curing agent (v/v) = 10:1) (Sylgard 184, Dow Corning) on a petri-dish at 1000 rpm for 1 min and curing at 70 °C for 2 h. A patterned 40 nm-thick Pt layer was formed by a shadow mask using sputtering (Intlvac Nanochrome AC/DC system). This Pt film on PDMS was mechanically stretched with 5% strain using a moving stage (THORLABS Model LTS150/M), and the cracks were formed in a highly controllable manner.

### Characterization of the resistive nanocracked Pt diaphragm sensor

To test the electro-mechanical responses of the nanocracked Pt sensor, a PDMS probe is attached to a force gauge (Mark-10 series 7-012) equipped to a test stand (Mark-10, ESM 301 L), the Pt sensor chamber together with a cardiac organoid was put on the test stage. Uniform pressure cycles were applied by a computer-based user interface, while the force and current readouts were measured by the force gauge and an electrochemical system (PARSTAT 4000 A, Princeton Applied Research) simultaneously.

### Integration of the force-sensing diaphragm into a soft culture well

The sensor chip was prepared following the steps shown in Fig. 1a. Firstly, a PDMS chamber mold was prepared by punching a hole at the central position of a PDMS base with a thickness of 0.5 cm. Then, the backside of the as-prepared cracked Pt film (the side without Pt metal) and the PDMS chamber mold were treated by oxygen plasma at 500 mTorr for 2 min. The two plasma treated layers were then placed together for chemical bonding. Next, the two ends of the outward cracked Pt film were connected with conductive threads (Adafruit) using liquid metal (Sigma-Aldrich) and silver paste (RS Components). The conjunction parts were further sealed by Eco-flex 35 (Smooth on Inc.) to ensure stable connections. Finally, the sensor chip was flipped over and a PDMS ring with a height of 1 cm was fixed on the sensor chip using Eco-flex 35. The prepared sensor chips were sterilized by 80% ethanol and UV exposure prior to culture and monitor of the cardiac organoids.

### AFM-like soft engagement design

A PDMS ellipsoid was fabricated by curing the PDMS precursors (base: curing agent (v/v) = 10:1) into a semi-ellipsoid mold (long side diameter = 16 mm, short side diameter = 12 mm, thickness = 5 mm) and curing at 70 °C for 2 h. Then the central area of the semi-ellipsoid was squeezed out using a puncher with 2 mm inner diameter. The soft PDMS probe was attached to a pin fixed into an *x*–*y*–*z* manipulator and was sterilized using 80% ethanol. Next, the sterilized PDMS probe was controlled to achieve optimal contact with the cardiac organoid in the bioreactor to ensure stable monitoring. The resistance changes of the cracked Pt strain sensor were real-time measured by an electrochemical workstation (PARSTAT 4000 A, Princeton Applied Research) at a sampling rate of 100 Hz.

The engaging process for the soft PDMS probe to contact with the cardiac organoid for real-time detection of beating events is referred to the design rationale of the AFM cantilever engaging process. Firstly, the target cardiac organoid together with culture media was transferred to the cracked Pt sensor chip. Controlled by the *x*–*y*–*z* manipulator, the soft PDMS probe approached towards the cardiac organoid. Because of the ultra-sensitivity of the cracked Pt sensor, each movement of the PDMS probe in the culture media was recorded by the connected electrochemical workstation. As the PDMS probe approached the cardiac organoid, the recorded periodic peaks caused by the beating organoid were stronger. However, after reaching a certain position, a further pressing could not induce stronger SNR indicating the cardiac organoid was over-touched. So subsequently, the soft PDMS probe was retracted, back to the previous position for optimal contact between the PDMS probe and the cardiac organoid. Hence, the soft PDMS probe was fixed at this position for following real-time detection.

### Electrical stimulation

Two Pt wires as the stimulation electrodes were parallelly inserted in the sensor chamber on opposite sides (9 mm apart). External electrical impulses (square wave pulses, 20 ms in width) at varied frequencies and varied electrical field strengths for different durations were generated using a function generator (AFG-2005, Gw Instek). After each stimulation period, the cardiac organoids were allowed at least 60 s to recover. Electrical stimulation was performed both to pace the cardiac organoids with spontaneous beating and to evoke spontaneous beating recovery from the temporarily non-beating cardiac organoids. The electrically paced cardiac contractions were simultaneously and continuously recorded by the electrochemical workstation providing real-time and instant readouts.

### Simultaneous recording of extracellular field potentials and mechanical contraction

Cardiac organoids were mounted on a 3D microelectrode array (60-3DMEA200/12/80iR-Ti-gr, Multichannel Systems, Germany) in cardiac organoid medium and recorded on a MEA1060-2BC system (Multichannel Systems) maintained at 37 °C. Recordings were taken at a sampling rate of 5 kHz and low-pass filtered using a 200 Hz Butterworth 2nd-order filter. The top-positioned diaphragm sensor was integrated as followed: firstly, a plastic tube was prepared by trimming a pipette tip. Then, the two ends of the outward cracked Pt film were connected with conductive threads (Adafruit) using liquid metal (Sigma-Aldrich). The conjunction parts were further encapsulated by adhesive film (Opsite Flexifix) to ensure stable connections. Next, the nanocracked Pt diaphragm sensor was stick to the prepared plastic tube. Finally, the top-positioned diaphragm sensor was stick to the *x*–*y*–*z* manipulator to control the position of the sensor. The prepared sensor was sterilized by 80% ethanol and UV exposure prior to its use.

### Dose-response studies

To test the responsiveness of cardiac organoids to chronotropic and inotropic agents, cardiac organoids were treated with different concentrations of carbachol by titration or media change method. A series of drug concentrations (from 1 nM to 10 μM) were freshly diluted from the stock solution prior to drug administration. For the titration method, a 4 μL drug drop was carefully added into the sensor chamber containing 200 μL organoid media. For the media exchange method, the 200 μL media in the sensor chamber was removed and replaced with 200 μL fresh media containing drug at a specific concentration. Before the dose-response study commenced, the cardiac organoids were equilibrated for 1 h following the setup of the system in the incubator. Cardiac contractility was then continuously measured throughout the whole process of cumulative drug dose administration and incubation.

### Statistical and reproducibility

Sample size was predetermined with a power calculation based on standard deviation of 10% observed in pilot experiments. A sample size of three experimental replicates was estimated to provide >80% power to detect at least a 22% change with ANOVA (SD = 10%, type I error set at *α* = 0.05 and type 2 error set at *β* = 0.2). Biological tissue samples were randomly allocated to treatment groups. Blinding was not performed in this study. Mechanical contractile analysis data were collected via an electrochemical workstation (PARSTAT 4000 A, Princeton Applied Research). Multi-electrode analysis data were collected on a MEA1060-2BC system (Multichannel Systems). For FACS data collection using FACS Diva Version 8.0.1. Video contraction data were analyzed using publicly available Image-J plugin MUSCLEMOTION v1.0. For FACS data analysis FLowJo v10.8 was used. For all other data processing and statistical analyses, GraphPad Prism 9.3.1 and OriginPro 20201b software were used. Data are presented as mean ± SEM, and a minimum of 3 organoids were used for statistical analysis with either a one-way ANOVA followed by a Bonferroni post hoc analysis or a Student’s *t*-test using GraphPad Prism 9.3.1. *P* < 0.05 was considered statistically significant. No data were excluded from the analysis. The rise time and decay time were analyzed and beat rate corrected with Fridericia’s formula^41^ (rise time corrected (Rc) = R/interspike interval^1/3^) and (decay time corrected (Dc) = D/interspike interval^1/3^), respectively. (RMSSD = $\sqrt{\frac{{\sum }_{i=1}^{N-1}{({{RR}}_{i}-{{RR}}_{i+1})}^{2}}{N-1}}$, where *N* is the number of total beats, *RR* represents the time difference between adjacent peaks).

### Reporting summary

Further information on research design is available in the Nature Portfolio Reporting Summary linked to this article.

## Supplementary information

Supplementary Information

Description of additional Supplementary File

Supplementary Video 1

Supplementary Video 2

Supplementary Movie 3

Supplementary Movie 4

Supplementary Movie 5

Supplementary Movie 6

Supplementary Movie 7

Reporting Summary

## Data Availability

All relevant data supporting the findings of this study are available within this article, its Supplementary Information files, or from the corresponding author upon reasonable request. Source data are provided with this paper.

## Source data

Source Data
