# Supplementary material for: A soft and ultrasensitive force sensing diaphragm for probing cardiac organoids instantaneously and wirelessly
Source: Nat Commun. 2022 Nov 25;13:7259. doi: 10.1038/s41467-022-34860-y (PMC9700778; doi:10.1038/s41467-022-34860-y)
Supplement: Supplementary file 1 — Supplementary Information [file 41467_2022_34860_MOESM1_ESM.pdf]

Supplementary Materials for

A Soft and Ultrasensitive Force Sensing Diaphragm for Probing Cardiac Organoids Instantaneously and Wirelessly

Quanxia Lyu[1,9], Shu Gong[1,9], Jarmon G. Lees[2,3,9], Jialiang Yin[1], Lim Wei Yap[1], Anne M. Kong[2], Qianqian Shi[1], Runfang Fu[1], Qiang Zhu[4], Ash Dyer[4], Jennifer M. Dyson[5,6], Shiang Y. Lim[2,3,7,8], Wenlong Cheng*[,1,4]

[1]*Department of Chemical & Biological Engineering, Monash University, Clayton, VIC, Australia.*

[2] *Department of Medicine and Surgery, University of Melbourne, VIC, Australia.*

[3] *O'Brien Institute Department, St. Vincent's Institute of Medical Research, VIC, Australia.*

[4] *The Melbourne Centre for Nanofabrication, Clayton, VIC, Australia.*

[5] *Department of Biochemistry & Molecular Biology, Biomedicine Discovery Institute, Clayton, VIC, Australia.*

[6] *Faculty of Engineering, Monash Institute of Medical Engineering (MIME), Monash University, Clayton, VIC, Australia.*

[7] *Drug Discovery Biology, Faculty of Pharmacy and Pharmaceutical Sciences, Monash University. Parkville, VIC, Australia.*

[8] *National Heart Research Institute Singapore, National Heart Centre, Singapore.*

[*] Correspondence author. Email: W. L. Cheng (wenlong.cheng@monash.edu)

[9] These authors contributed equally to this work.

Table of Contents

Section I Fabrication and characterization of cracked Pt sensing diaphragm **4**

I-1. Fabrication of the nanocracked Pt sensing diaphragm 4

I-2. Definition of signal/noise ratio (SNR) of the beating patterns 6

I-3. Performance characterization of the nanocracked Pt sensing diaphragm 9

Section II Real-time continual monitoring of the dynamic beatings of cardiac organoids **15**

Section III Validation and simultaneous multi-modal measurement **17**

III-1. Simultaneous video measurement 17

III-2. Simultaneous electrophysiological measurement 22

Section IV Real-time monitoring of cardiac contractions during electrical stimulation (ES) **23**

IV-1. Deriving the relationship between the electrical readout and the straining energy (E_B) 24

IV-2. Influence of ES duration on the cardiac organoid at room temperature 27

IV-3. Influence of ES field strength on the cardiac organoid at room temperature 29

IV-4. Monitoring subtle changes of organoid beating patterns during resuscitation 31

IV-5. Influence of ES field strength on pacing the resuscitated cardiac organoid 33

IV-6. Influence of ES frequency on pacing the resuscitated cardiac organoid 35

IV-7. Influence of ES duration on pacing the resuscitated cardiac organoid 36

IV-8. Comparison of heart rate variability (RMSSD) of a healthy organoid and a resuscitated organoid 37

Section V Monitoring cardiac organoid contractility during drug dosing **38**

V-1. Carbachol dose-response studies 39

V-2. ES on the carbachol-treated cardiac organoid 45

V-3. Administration of carbachol via media exchange method 47

Section VI FACS gating strategy for the vascularised cardiac organoids **49**

List of Supplementary Videos Error! Bookmark not defined.

Inspired by the mechanotransduction process in a wrist pulse diagnosis, we report an E-skin-based, conformal soft sensor system (Fig. S1). In the pulse diagnostics, a pulsing vein is sandwiching between a patient's tissue and a doctor fingertip, which is essentially an all-soft-contact mechanotransduction process requiring optimal positioning and holding forces so that the doctor brain can detect minute strain changes related to systolic and diastolic function of the human heart (Fig. S1a). Analogously, in a cardiac organoid diagnostic, the organoid is sandwiched between two soft PDMS "fingers" in order to achieve soft conformal microcontact (Fig. S1b). The bottom "finger" is PDMS encapsulated nanocracked platinum (Pt) E-skin mimicking the function of mechanoreceptors of fingertip; whereas the top "finger" is a PMDS semi-ellipsoid controlled by x-y-z positioner. This setup offers a simple yet efficient strategy to establish conformal E-skin soft sensor-organoid microcontact – crucial for the reliable detection of cardiac organoid beating in real-time and *in situ*.

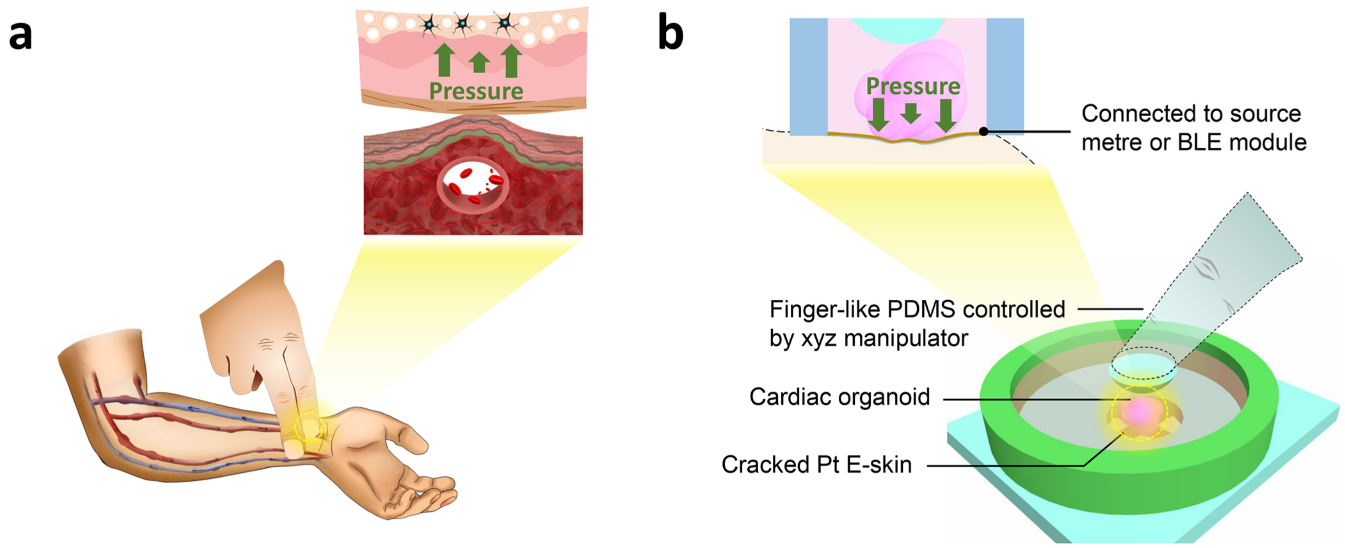

Fig. S1 (**a**) Schematic of the pulse diagnose process in traditional Chinese medicine. (**b**) Schematic of the soft tactile finger sensing system inspired by the finger-pulsing process.

Section I Fabrication and characterization of cracked Pt sensing diaphragm

I-1. Fabrication of the nanocracked Pt sensing diaphragm

The fabrication process of the nanocracked Pt sensing diaphragm are demonstrated in Figure 1a. Firstly, a 100 μm thick PDMS layer was fabricated by spin-coating PDMS precursors (base: curing agent (v/v) =10:1) on a petri-dish at 1,000 rpm for 1 minute and curing at 70℃ for 2 hours. A patterned 40 nm-thick Pt layer was deposited by a shadow mask (Fig. S1) using sputtering (Intlvac Nanochrome AC/DC system). This Pt film on PDMS was mechanically stretched at 5% strain for 10 cycles using a moving stage (THORLABS Model LTS150/M), and the cracks are formed in a highly controllable manner. The cracked Pt film was further bonded on a PDMS mold with a hole in the center by air plasma treatment for 2 minutes. The cracked Pt film was then connected with the conductive thread and the joint places were sealed by Eco-flex 35 (Smooth On, Inc). Then a PDMS culture chamber was bonded on the PDMS mold by air plasma treatment for 2 minutes and then further sealed by Eco-flex 35.

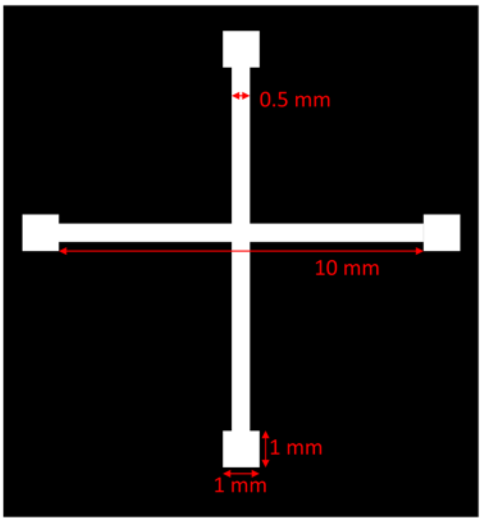

Fig. S2 The dimension of the shadow mask for cracked Pt sensing diaphragm.

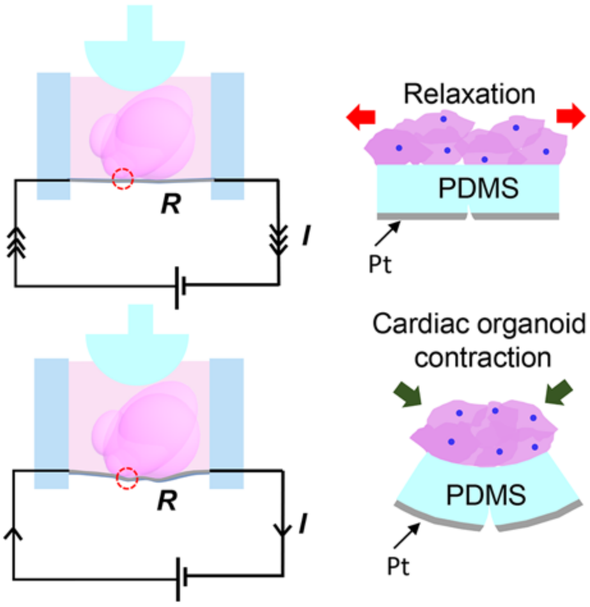

Fig. S3 The operation principle of the piezoresistive cracked Pt-based diaphragm to detect the beating of the cardiac organoid. Under optimal contact condition, the crack gap increases with the contraction of cardiac organoid, leading to reduced electrical conductivity of the diaphragm.

I-2. Definition of signal/noise ratio (SNR) of the beating patterns

The definition of SNR is the ratio of mean to standard deviation of a signal:

$$SNR = \frac{\mu}{\sigma} \qquad (1)$$

where μ is the mean value of the amplitude of each beating patterns, σ is the standard deviation of the noise (Fig. S4). The real-time force changes under different contact states are measured in Fig. S5 using a Mark-10 force gauge (M7-012). The calculated SNR with different compressive force is summarized in Fig. S6, indicating a soft conformal contact between the organoid and PDMS probe could lead to a large SNR of over 10 times than other states.

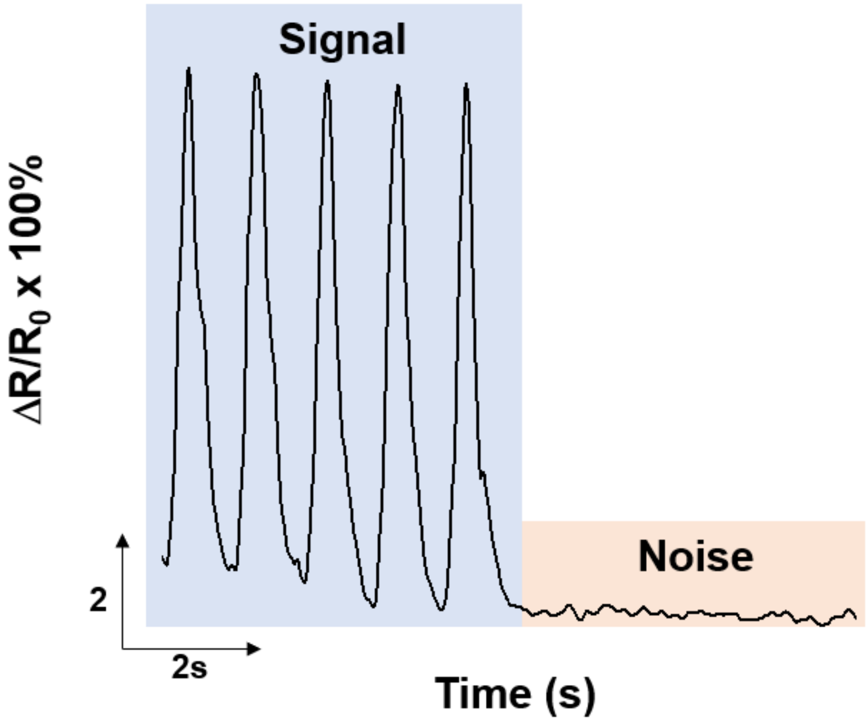

Fig. S4 An example of the signal and noise of the resistance changes of the nanocraked Pt sensor in response to beating events.

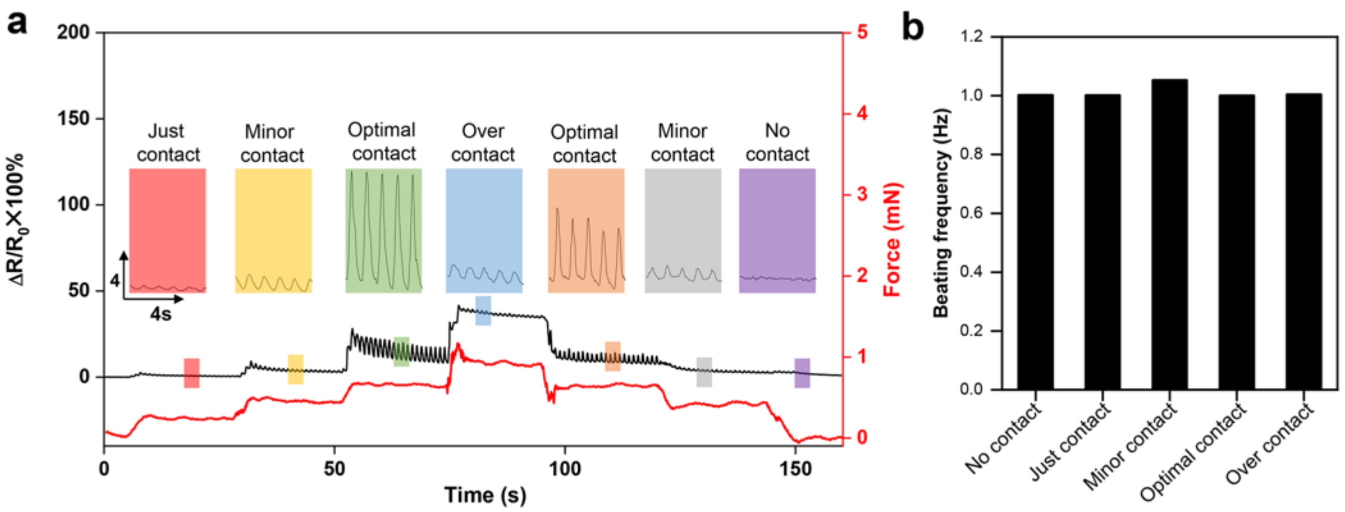

Fig. S5 a Relationship between the resistance changes of the diaphragm sensor and the force changes of the force gauge under different contact conditions. **b** Comparison of beating frequency of an organoid under different contact conditions. These results indicate that using a soft PDMS probe to push against the organoid induces minimal alterations in the organoid's functionality.

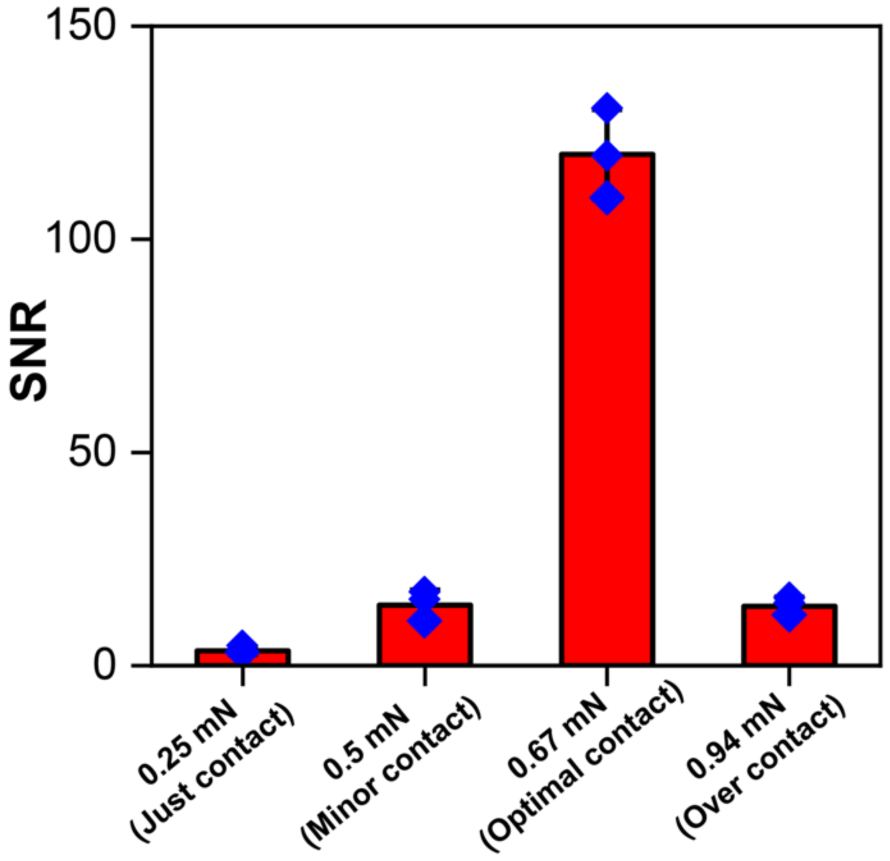

Fig. S6 The calculated SNR of the beating pattern with different compressive force by the PDMS probe (n=3). Data are presented as mean ± SEM.

I-3. Performance characterization of the nanocracked Pt sensing diaphragm

The PDMS probe was attached to a force gauge (Mark-10 M7-012) controlled by a testing stand (Mark-10 ESM 301). A non-beating cardiac organoid was put on the sensing diaphragm surrounded by a soft PDMS culture chamber. A PDMS cylindrical probe (2 mm in diameter, 5 mm in thickness) was controlled to periodically contact with the cardiac organoid to mimic the beating events of a cardiac organoid. The force gauge was used to record the applied pressure and the current changes of the sensing diaphragm is recorded by an electrochemical workstation (PARSTAT 4000A, Princeton Applied Research). The experiment set-up is demonstrated in Fig. S7a. We evaluated the sensitivity of the pressure sensor by defining the sensitivity *S*:

$$S = \frac{\Delta I}{I_0 \Delta P} \qquad (2)$$

where $\Delta I = I - I_0$ is the current difference with loading and unloading of the pressure, and ΔP is the difference in applied pressure. We estimated the pressure by defining the area of the PDMS probe as the contacted area. The test is performance under optimal contact condition. The sensitivity of the nanocracked Pt sensing diaphragm is 5.64 kPa^{-1} from 0-160 Pa and increased to 38.2 kPa^{-1} from 160-250 Pa. Each region showed good linearity between the current changes and pressure (Fig. S7b). We evaluated the gauge factor of the diaphragm sensor by defining the *GF*:

$$GF = \frac{\Delta R}{R_0 \varepsilon} \qquad (3)$$

where $\Delta R = R - R_0$ is the resistance difference with stretched and relexed strain, and ε is the difference in applied strain. The gauge factor of the diaphragm is 338.9 in the specific organoid beating range (Fig. S8).

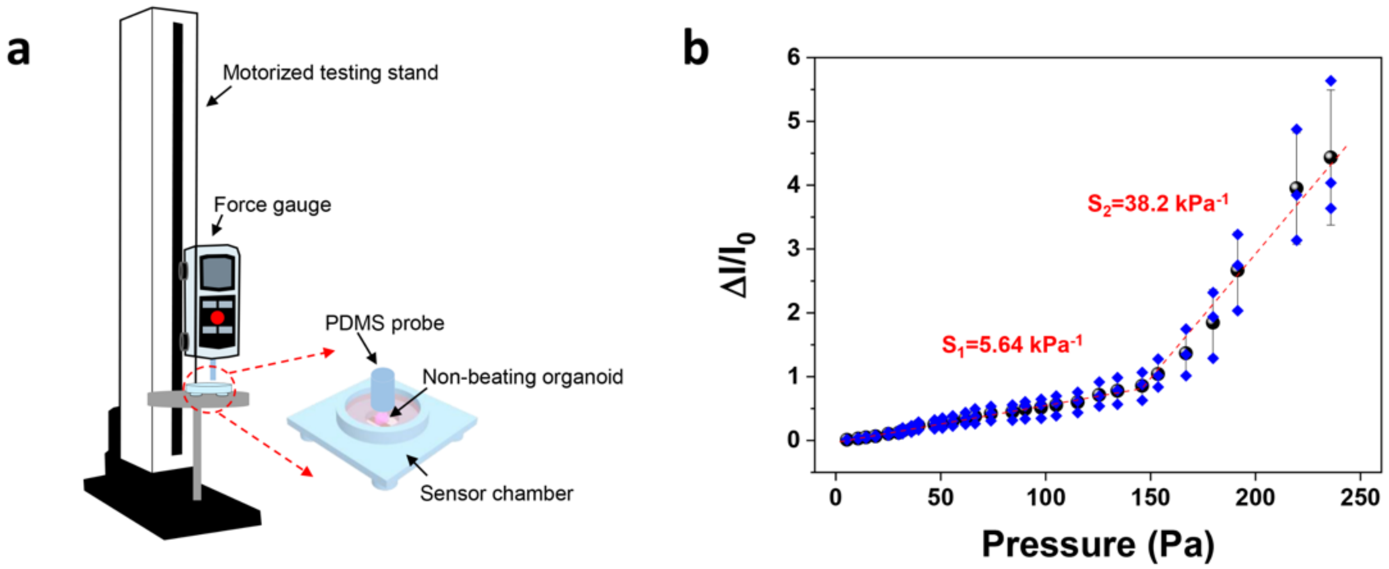

Fig. S7 a Experiment set-up for the characterization of pressure and force sensitivity of the diaphragm sensor. **b** Relationship between applied pressure and current changes of the diaphragm sensor when a non-beating cardiac organoid is attached on the diaphragm. Three sensors were tested in the experiment (n=3). All the test was performed in the room temperature (25°C). Data are presented as mean ± SEM.

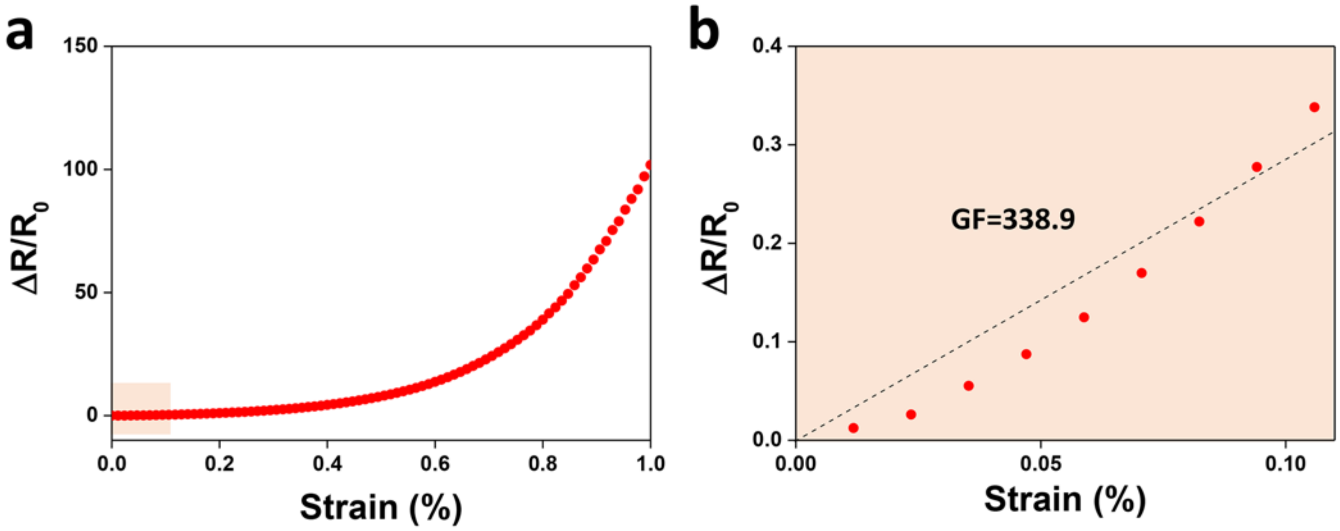

Fig. S8 a Resistance changes as a function of strain of the diaphragm sensor in the strain range of 0-1%. **b** Gauge factor of the diaphragm sensor in the organoid beating force range.

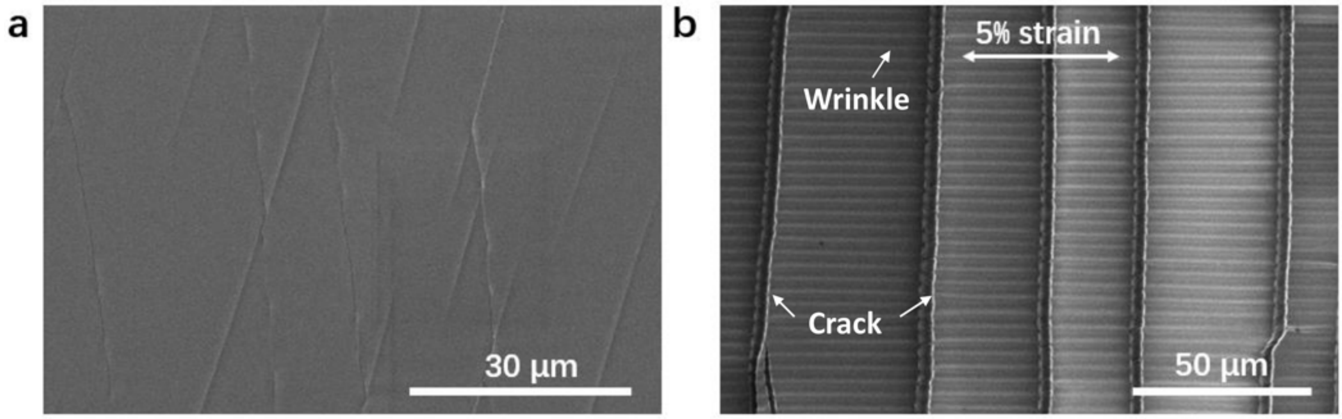

Fig. S9 Scanning electron microscope (SEM) images of the cracked Pt film on PDMS with no strain (**a**) and 5% tensile strain (**b**) applied. **a** and **b** are representative SEM images from three independent experiments with similar results.

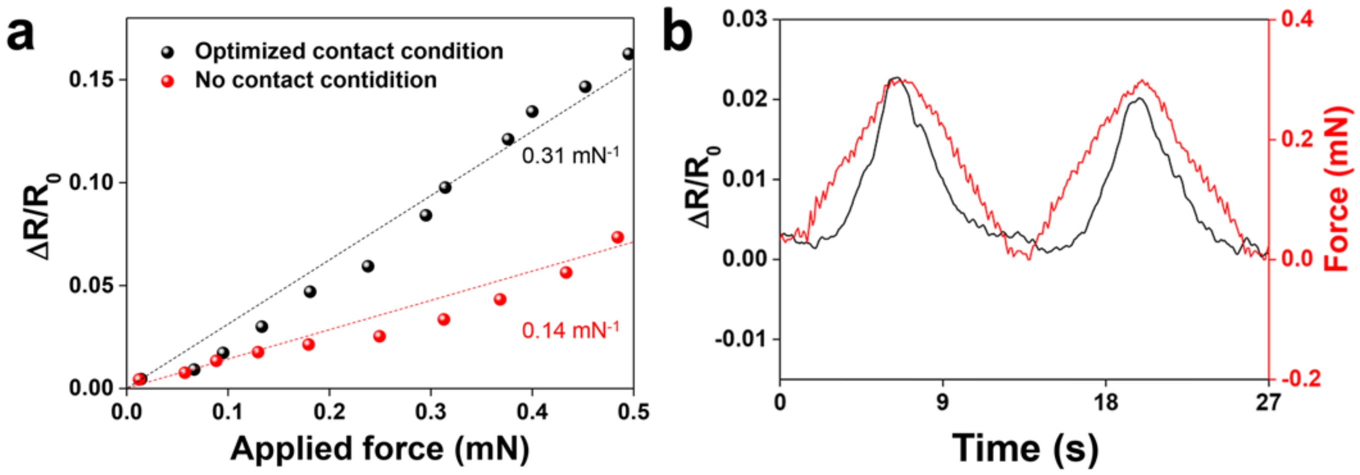

Fig. S10 a Relationship between applied force and sensor resistance ratio under “optimal contact” and “no contact” conditions. **b** Dynamic force changes with sensor resistance changes under ‘no contact’ condition.

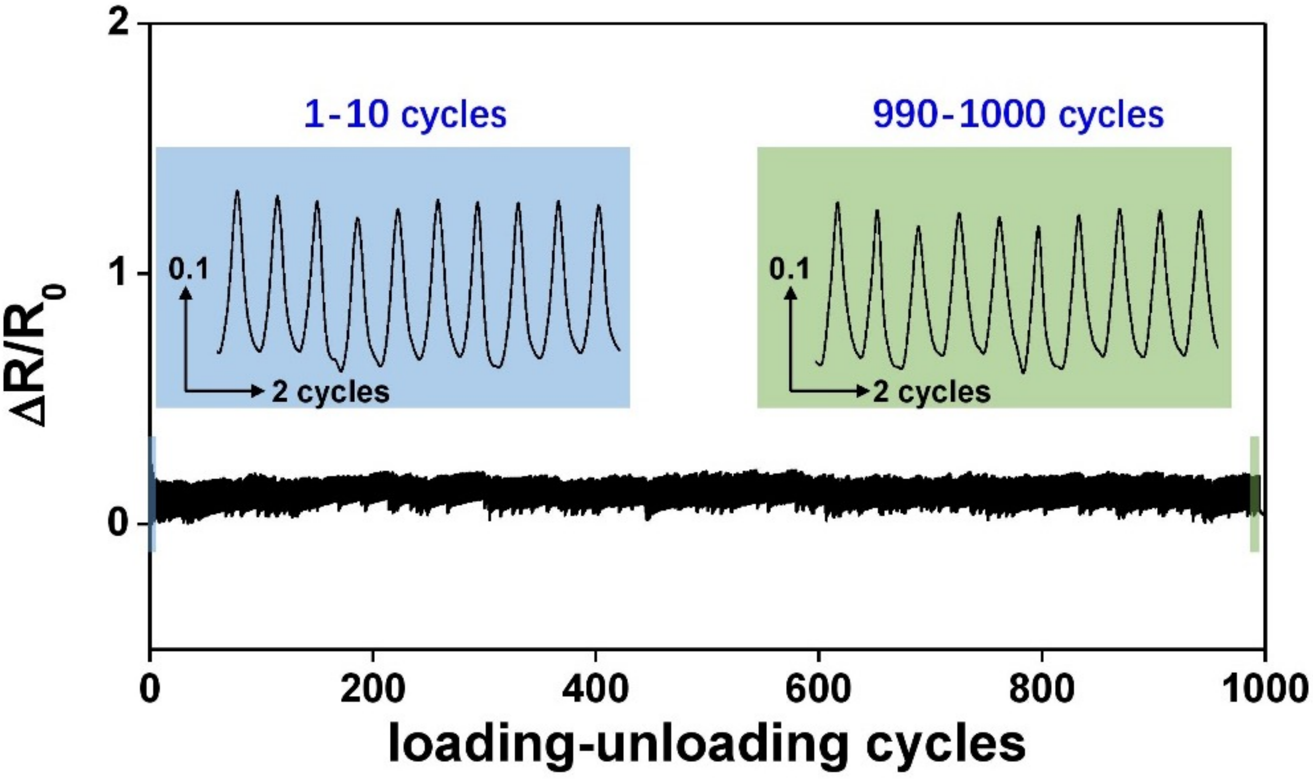

Fig. S11 The performance of the nanocracked Pt sensor towards 1,000 repeated loading-unloading cycles at a dynamic force from 0-0.5 mN.

Section II Real-time continual monitoring of the dynamic beatings of cardiac organoids

The sterilized sensing diaphragm-integrated culture chamber was put into an incubator at 37ºC with 5% CO_2 and 95% humidity. A beating cardiac organoid was transferred into the chamber containing 200 μL of organoid medium for contractile assessment. A PDMS soft probe was fixed in the incubator to maintain a conformal contact between the nanocracked Pt sensing diaphragm and organoid. The changes in electrical resistance of the sensor were recorded by an electrochemical workstation continuously for 7 hours while the beating frequency of the organoid was recorded by using a MATLAB program based on the response of the diaphragm sensor.

Fig. S12 a-g shows the real-time changes in contractile profile monitored by the miniaturized sensor chip inside a 37ºC humidified CO_2 incubator for 7 hours. During the first hour of culture, a clear increase in spontaneous beating frequency was recorded, which may correspond to an acclimatization to the optimal culture environment inside the 37ºC humidified CO_2 incubator. In the following 1-3 hours period of culture, the spontaneous averaged beating rate remained stable at 2.3 Hz with occasional fluctuations of beating frequency. In the following hours of culture, the spontaneous beating rate steadily and slowly decreased without many fluctuations (Fig. S12h). After 5 hours of culture, the relative beating force decreased to half the initial level (Fig. S12i). Also, the fluctuation in spontaneous beating rate was apparent in the 6-7 hours culture period (Fig. S12j). These fluctuations and decrease in relative beating force may have been caused by a reduction in nutrient levels or pH changes of the culture media due to the high metabolism of the beating cardiac organoid.

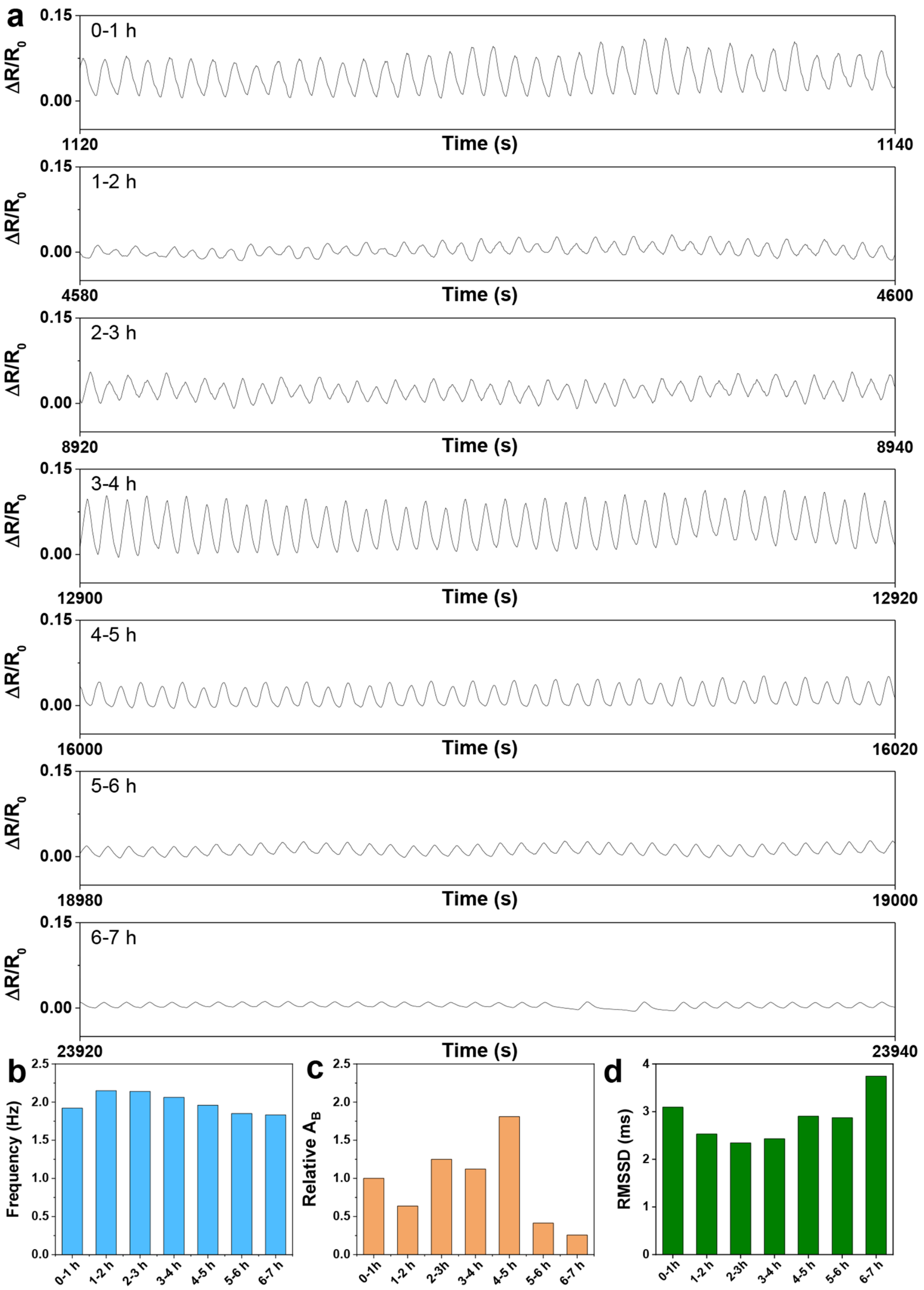

Fig. S12 a-g A representative real-time resistance ratio change of the diaphragm sensor in response to an engineered beating cardiac organoid over a culture period of 7 hours in an incubator at 37°C with 5% CO_2 and 95 % humidity. **h** Changes in averaged beating frequency of the organoid measured hourly. **i** Changes in relative contract force of the organoid measured hourly. **j** The root mean square of the differences of successive R-R interval (RMSSD) (ms) measured hourly. Data are presented as mean ± SEM.

Section III Validation and simultaneous multi-modal measurement

III-1. Simultaneous video measurement

An organoid cultured in the diaphragm sensor chamber was put in a humidified CO_2 incubator together with a digital microscope (Dino-lite AM73915MZTL) and a lamp (Figure 2a). Optimal contact condition was pre-adjusted by a x-y-z manipulator. The elapsed time of optical and force signals was aligned through post-data analysis.

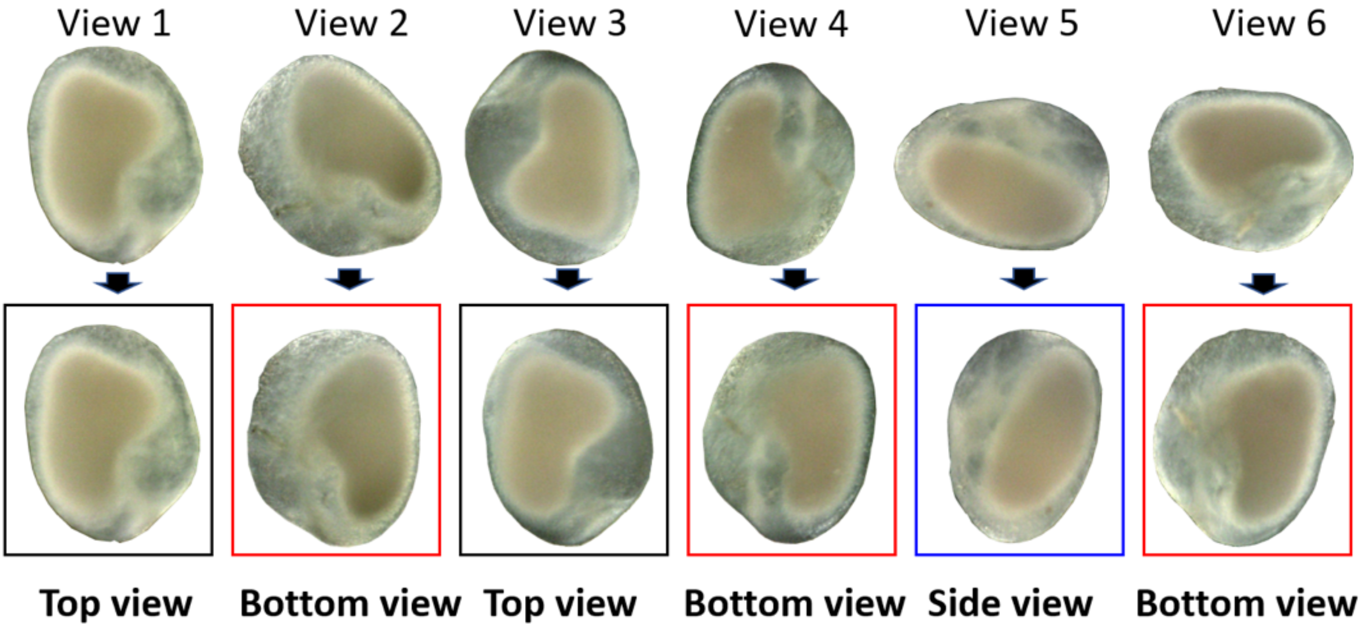

Fig. S13 Screenshot of 6 video recordings containing top view, bottom view and side view of the same organoid.

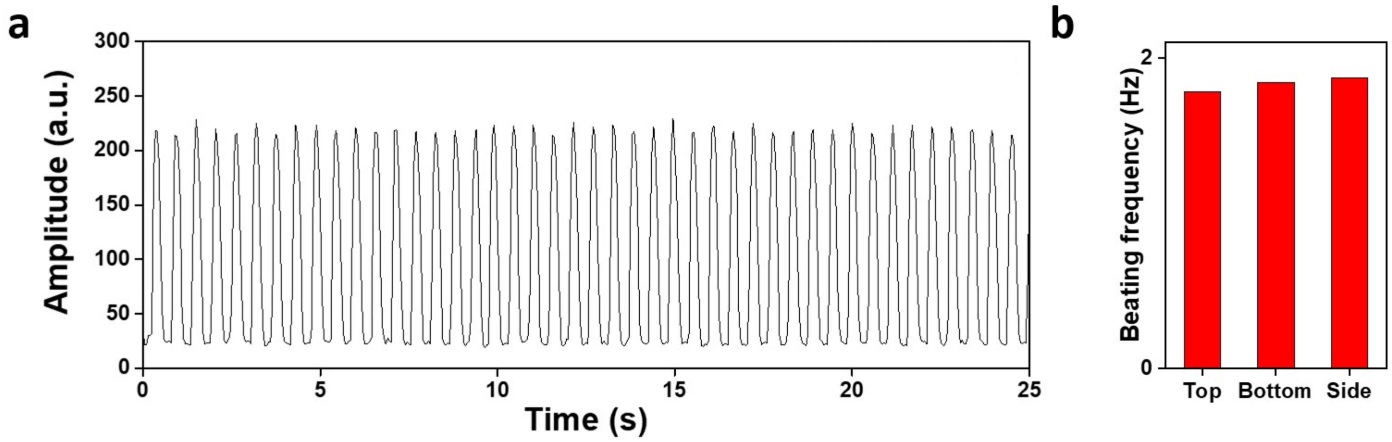

Fig. S14 a An example of video analysis result from a bottom view organoid beating recording. **b** Bar plot showing the beating frequency of the same organoid based on video analysis.

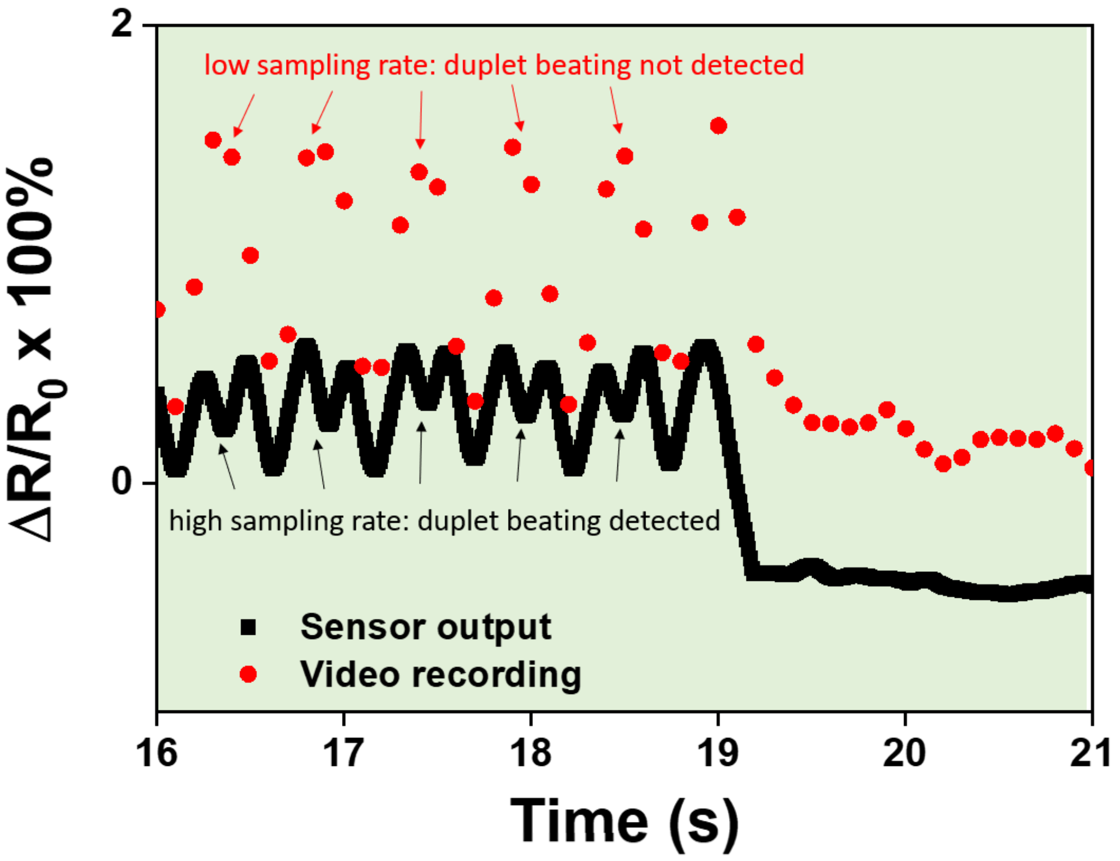

Fig. S15 Comparison of the sensor output and video recording towards fine details of the organoid beating.

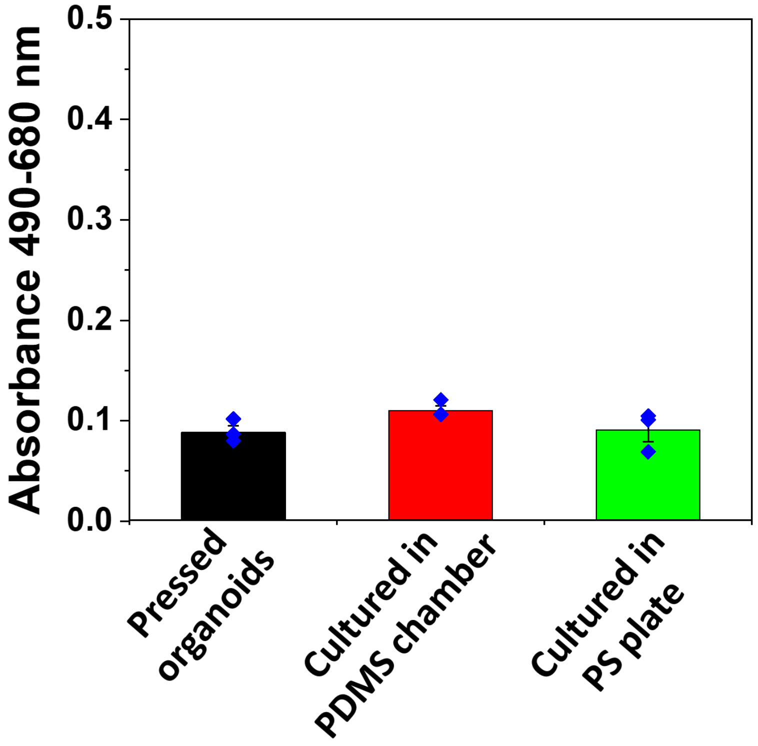

Fig. S16 Lactate dehydrogenase (LDH) test for organoids cultured for 24 h in a polystyrene (PS) plate, PDMS chamber, and PDMS chamber pressed by PDMS probe for 2 h (n=3). Data are presented as mean ± SEM.

III-2. Simultaneous electrophysiological measurement

An organoid was transferred to a commercial microelectrode array (MEA, multichannel systems, 60-3DMEA200/12/50iR-Ti-gr) device. The culture medium temperature was maintained at 37°C. A top-probe-based diaphragm sensor was located above the MEA chamber (Figure 2g) adjusted by a x-y-z manipulator. Optimal contact condition was achieved before the simultaneous electrophysiological and force measurement. The elapsed time of electrophysiological and force signals was aligned through post-data analysis.

In the carbachol dosage experiments, the beating of organoids was recorded for 30 minutes: 0-10 minutes' data was recorded for organoids before drug treatment, followed by carbachol dosage (1 μM) from 10-20 minutes, and carbachol dosage (10 μM) from 20-30 minutes. The beating frequency, relative first peak amplitude, and relative beating force of each organoid (n=3) were collected and compared at 5 minutes (0 μM carbachol), 15 minutes (1 μM carbachol), and 25 minutes (10 μM carbachol).

Section IV Real-time monitoring of cardiac contractions during electrical stimulation (ES)

Two Pt wires as the stimulation electrodes were parallelly inserted in the sensing diaphragm-integrated culture chamber on the opposite sides (9 mm apart). External electrical impulses (square wave pulses, 20 ms pulse width) at varied frequencies and varied electrical field potentials for different durations were generated using a function generator (AFG-2005, Gw Instek). After each stimulation period, the cardiac organoids were allowed to rest for at least 60 seconds. ES was performed either to pace the cardiac organoids that exhibited spontaneous beating activity and to evoke recovery of spontaneous beating activity from non-beating cardiac organoids. The electrically paced cardiac contractions were simultaneously and continuously recorded by the electrochemical workstation resulting in a real-time readout.

IV-1. Deriving the relationship between the electrical readout and the straining energy (E_B)

We developed a 'balloon' model to correlate the electrical line profiles of beating patterns to the strain energy consumed in each beating event. In the relax state $t=t_0$ (Fig. S17), a pressure equilibrium is established throughout the sensing diaphragm/organoid interface. Upon beating at time t, the sensing diaphragm deforms conformally to the contracting organoid tissue due to the soft intimate contact established in our system.

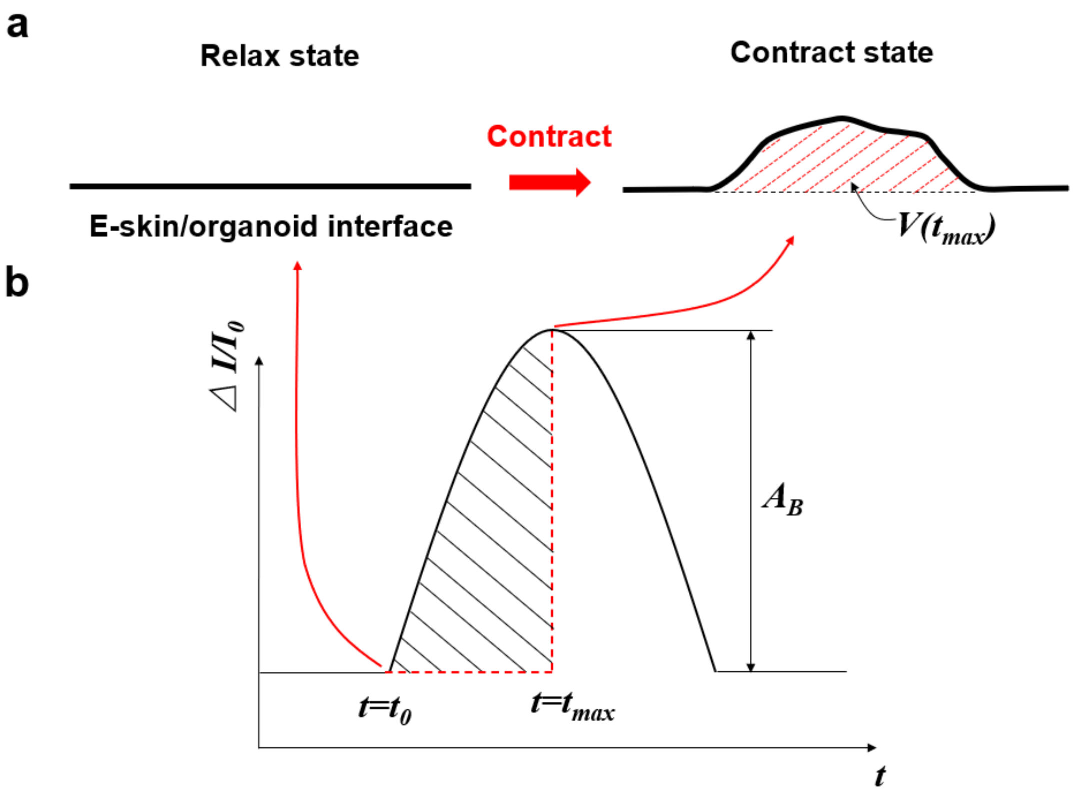

Fig. S17 a Scheme of the cross-section sensing diaphragm/organoid interface in the relax state and contract state. **b** The corresponding beating pattern in this process.

The degree of the sensing diaphragm deformation is dependent on the dynamic local pressure difference $\Delta P(t)$ (Fig. S18). With differential sensing diaphragm displacement dl, the differential amount of work (dW_s) done on the sensing diaphragm can be estimated by:

$$dW_s = \Delta P(t)A(t)dl = \Delta P(t)dV(t) \qquad (4)$$

where $A(t)$ is the dynamic sensing diaphragm/organoid contacting area.

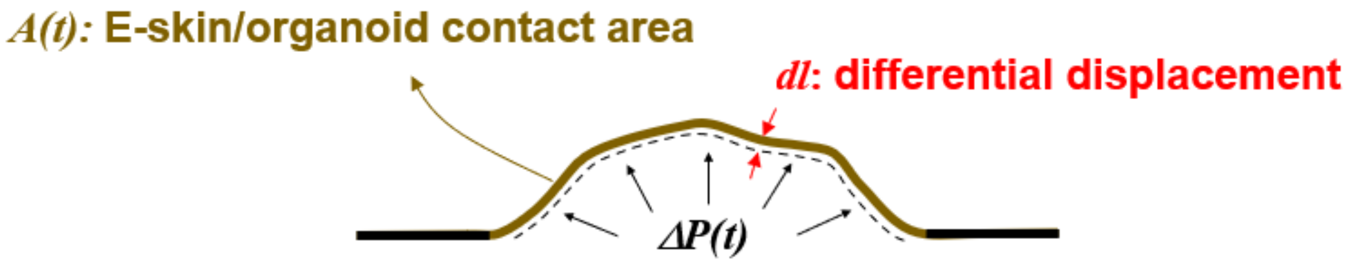

Fig. S18 Scheme of the 'balloon' model.

Integrate equation (3) from the fully relaxed state to the maximum contracted state (Fig. S18):

$$W_s = \int_0^{Vmax} \Delta P(t)dV(t) \qquad (5)$$

Based on equation (2), we know:

$$\Delta P(t) = \frac{\Delta I}{I_0 S} \qquad (6)$$

substitute equation (5) into equation (4):

$$W_s = \int_0^{Vmax} \frac{\Delta I}{I_0 S} dV(t) \qquad (7)$$

Assuming the uniform rate of sensing diaphragm strained by the organoid, then the volumetric deformation rate R^* is:

$$R^* = \frac{dV(t)}{dt} \qquad (8)$$

Plug equation (7) into equation (6), we have:

$$W_s = R^* \int_{t_0}^{t_{max}} \frac{\Delta I}{I_0 S} dt \qquad (9)$$

As shown in Figure 1f, the sensitivity *S* in the organoid beating force range (0-0.5 mN) is constant, hence, during the organoid relax-contraction process, we have:

$$W_s = \frac{R^*}{S} \int_{t_0}^{t_{max}} \frac{\Delta I}{I_0} dt \qquad (10)$$

This equation implies that the straining work done by the organoid on the sensing diaphragm is proportional to the first half of the Gaussian peak area in *i-t* curve recorded in our experiment (Fig. S17b). Therefore, it also predicts that the strain energy of the organoid associated with each beating event is also proportional to the first half of the Gaussian peak area.

For a certain time period *T* with *n* peaks, the energy consumed from straining events per second ($\overline{E_B}$) will be:

$$\overline{E_B} \propto \frac{1}{T} \sum_{i=1}^{i=n} \int_{t_i^0}^{t_i^{max}} \frac{\Delta I}{I_0} dt \qquad (11)$$

In this study, we select a specific time period T=15s for the calculation of normalized $\overline{E_B}$ in each state to compare their energy consumption.

In addition, the Gaussian peak amplitude A_B in Fig. S18 is directly related to the maximum contractile force F_s. As shown in Fig. S17, the relative current linearly increases with the force applied. The gradient *k* is estimated to be 0.31 mN^{-1}, hence, the maximum contact force for each beating event is:

$$F_s = \frac{\Delta I / I_0}{k} = \frac{A_B}{k} = 3.1 A_B \qquad (12)$$

The estimated contractile force F_s and normalized $\overline{E_B}$ is summarized in Fig. 3c.

IV-2. Influence of ES duration on the cardiac organoid at room temperature

The influence of ES duration on the cardiac organoid was investigated (Fig. S19) by fixing f_S=1 Hz and V_S=0.2 V mm^{-1} The time-lapsed real-time beating pattern demonstrated synchronization of cardiac organoid beating frequency with the stimulation frequency regardless of stimulation duration. The recovery time was also increased from ~1.1 seconds to ~9.7 seconds when t_S increased from 15 seconds to 120 seconds Interestingly, both F_s and $\overline{E_B}$ for post-ES increased substantially with >90s stimulation (Fig. S19c), indicating the importance of ES duration for cardiac organoids to establish enhanced post-ES calcium cycling for improved electromechanical function.

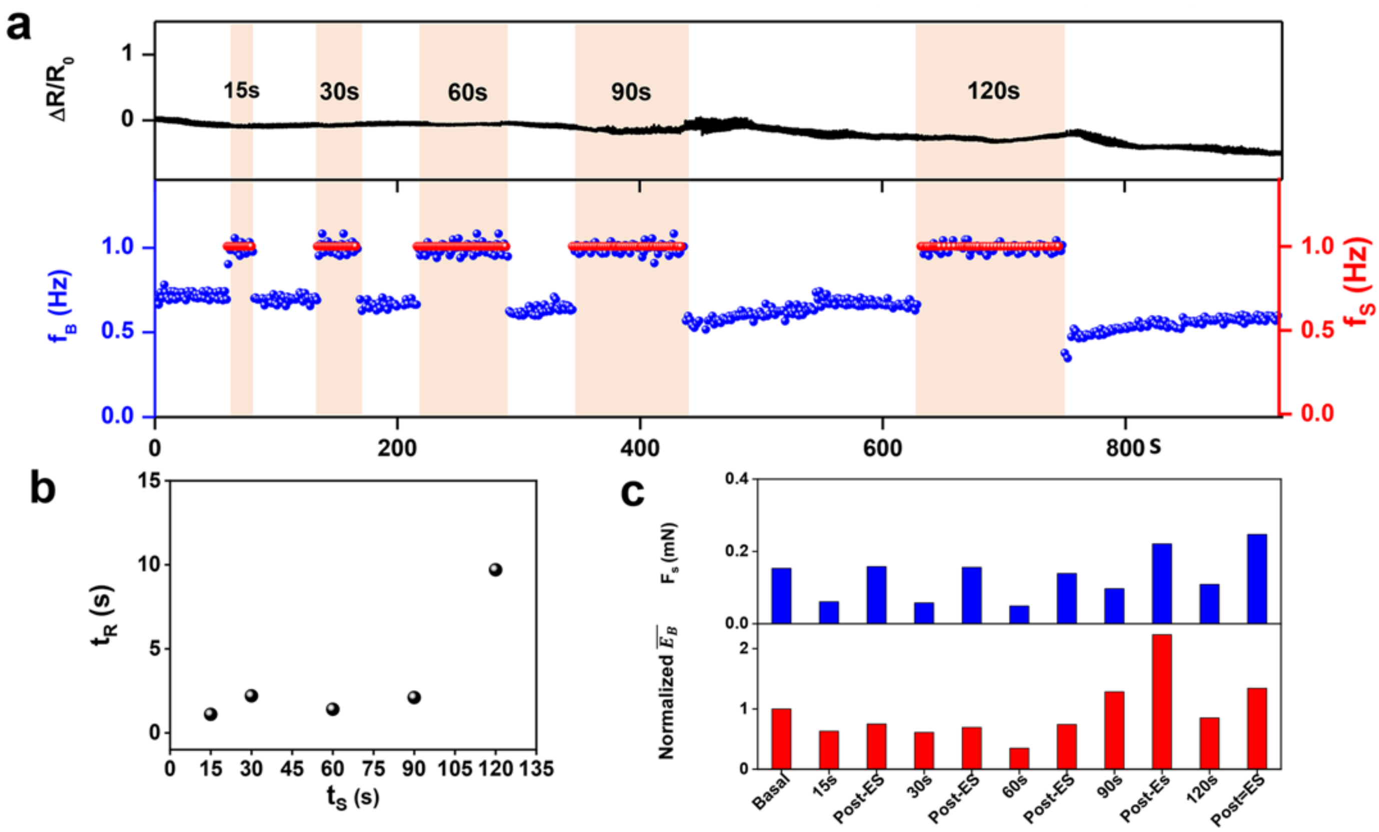

Fig. S19 a Real-time resistance change (top) and beating frequency (blue dot, bottom) during periods of electrical stimulation (red dot, bottom) with different ES times. left to right: 15s, 30s, 60s, 90s and 120s with a field potential of 0.2V mm $^{-1}$. **b** Comparison of the recovery time after each ES was removed. **c** The calculated contractile force (F_s) and normalized energy consumed from straining events per second ($\overline{E_B}$) of each period for a single organoid.

IV-3. Influence of ES field strength on the cardiac organoid at room temperature

The influence of ES field strength on the cardiac organoid was investigated (Fig. S20a). The time-lapsed real-time beating pattern demonstrated synchronization of organoid beating frequency and stimulation frequency with applied field strengths of 0.2 V mm^{-1} and 0.3 V mm^{-1} but not at an applied field strength of 0.1 V mm^{-1} (Fig. S20b). The beating amplitude reduced as the stimulation field strength increased (Fig. S20c). In addition, a 'double peaks' beating pattern with alternate smaller beating amplitudes was observed during ES (insert of Fig. S20a). The averaged amplitude (A_B) and the straining energy (E_B) presented in the manuscript was calculated over 10 consecutive beats.

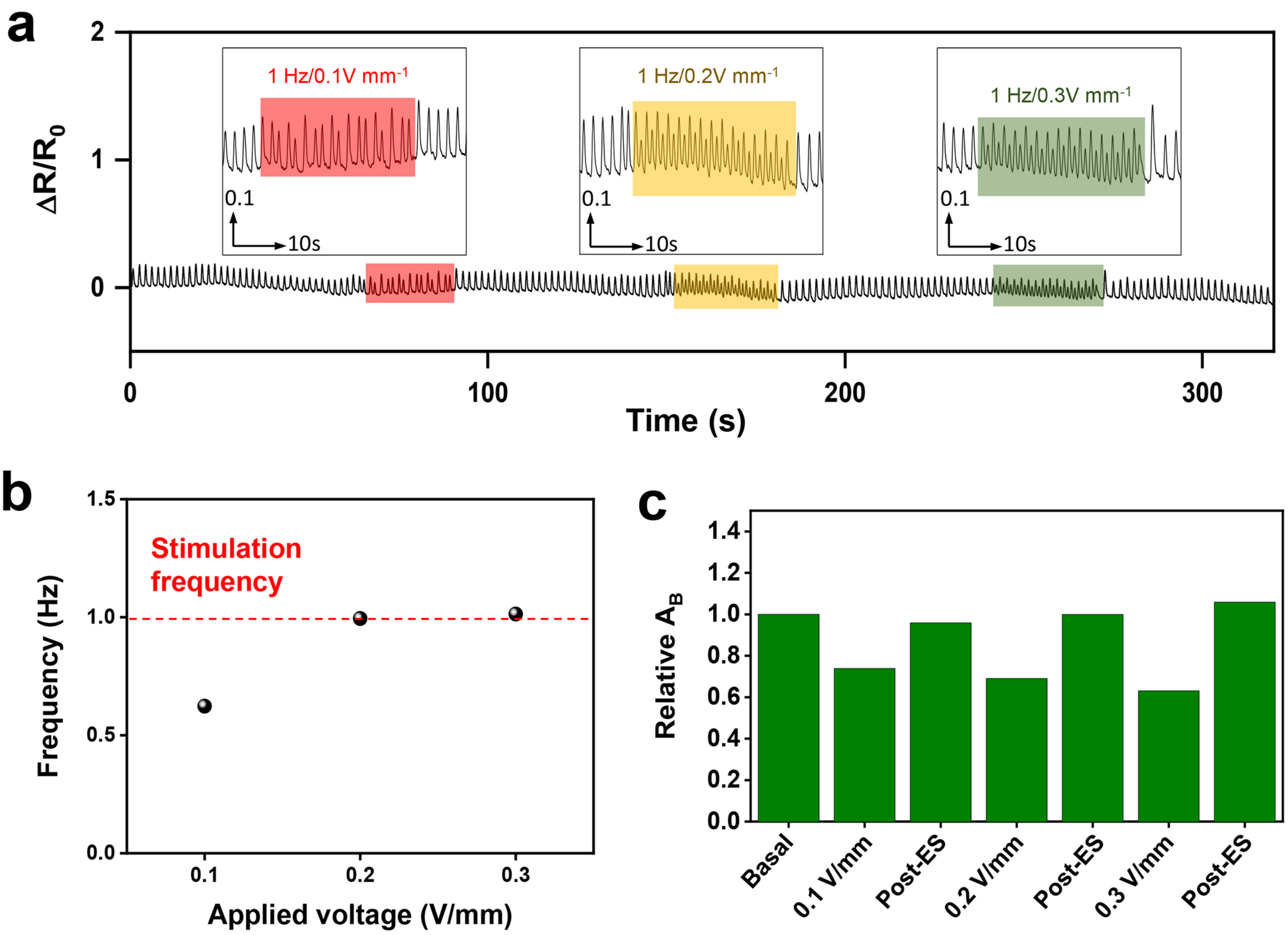

Fig. S20 a Real-time resistance change of the diaphragm sensor towards a period of ES with different ES field strength. left to right: 0.1 V mm^{-1}, 0.2 V mm^{-1}, and 0.3 V mm^{-1}. Waveforms in color shadings are organoid beatings during ES. **b** The average beating frequency during ES with different stimulation field strengths. **c** The relative contract force during ES with different stimulation field strengths. Data are presented as mean ± SEM.

IV-4. Monitoring subtle changes of organoid beating patterns during resuscitation

The soft nanocracked Pt sensing diaphragm could also accurately identify the dynamic beating patterns during an electrical resuscitation process for cardiac organoids that had lost their spontaneous beating activity after prolonged culture (Fig. S21a, b). While a non-beating cardiac organoid can be paced at an applied frequency (f_S=1 Hz, t_S=30 seconds), no spontaneous beating activity was observed upon the withdrawal of applied ES even when the stimulation duration was increased up to 120 seconds. However, ES at 2 Hz for 60 seconds was able to resuscitate the spontaneous beating activity of the cardiac organoid. The close-up view shows that the resuscitated cardiac organoid exhibited a slow, irregular beating frequency upon removal of ES, but gradually established a regular beating pattern after about 71 seconds (f_B=~0.5Hz).

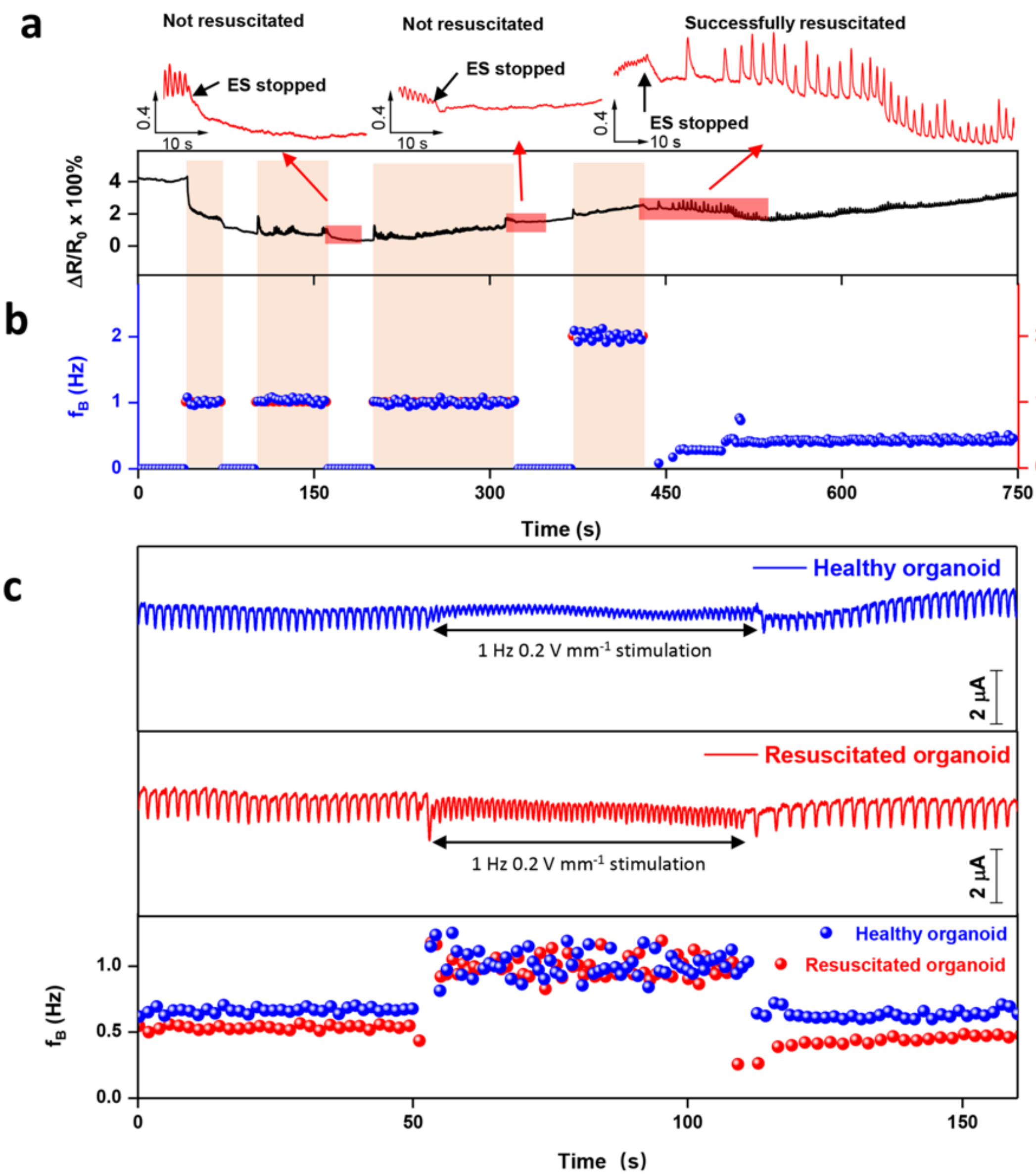

Fig. S21 The dynamic process for a non-beating cardiac organoid to recover spontaneous beating paced by electrical impulses. **a** The real-time detection and instant readouts of the whole process to resuscitate the cardiac organoid that has lost spontaneous beating activity by electrical stimulation at 0.2 V mm^{-1}. Insets are enlarged views of beating patterns during and post-electrical stimulation. Waveforms in red color shadings are organoid beatings during ES and right after ES. **b** The detailed patterns of the applied electrical stimulation frequency (red dot) and the measured real-time beating frequency (blue dot) of the cardiac organoid. **c** Comparison of the responses to 1 Hz electrical stimulation at 0.2 V mm^{-1} for 60 s from the healthy and the resuscitated cardiac organoids.

IV-5. Influence of ES field strength on pacing the resuscitated cardiac organoid

The influence of ES field strength on the resuscitated cardiac organoid is shown in Fig. S22. The real-time beating pattern showed synchronized beating frequency towards stimulation frequency with applied field strength from 0.1 to 0.3 V mm^{-1} (Fig. S22a). Furthermore, the organoid took a longer time to resume spontaneous beating activity post-stimulation with a higher applied field strength (insert of Fig. S22a). Similar to the healthy organoid, the beating amplitude reduced as ES field strength increased (Fig. S22c).

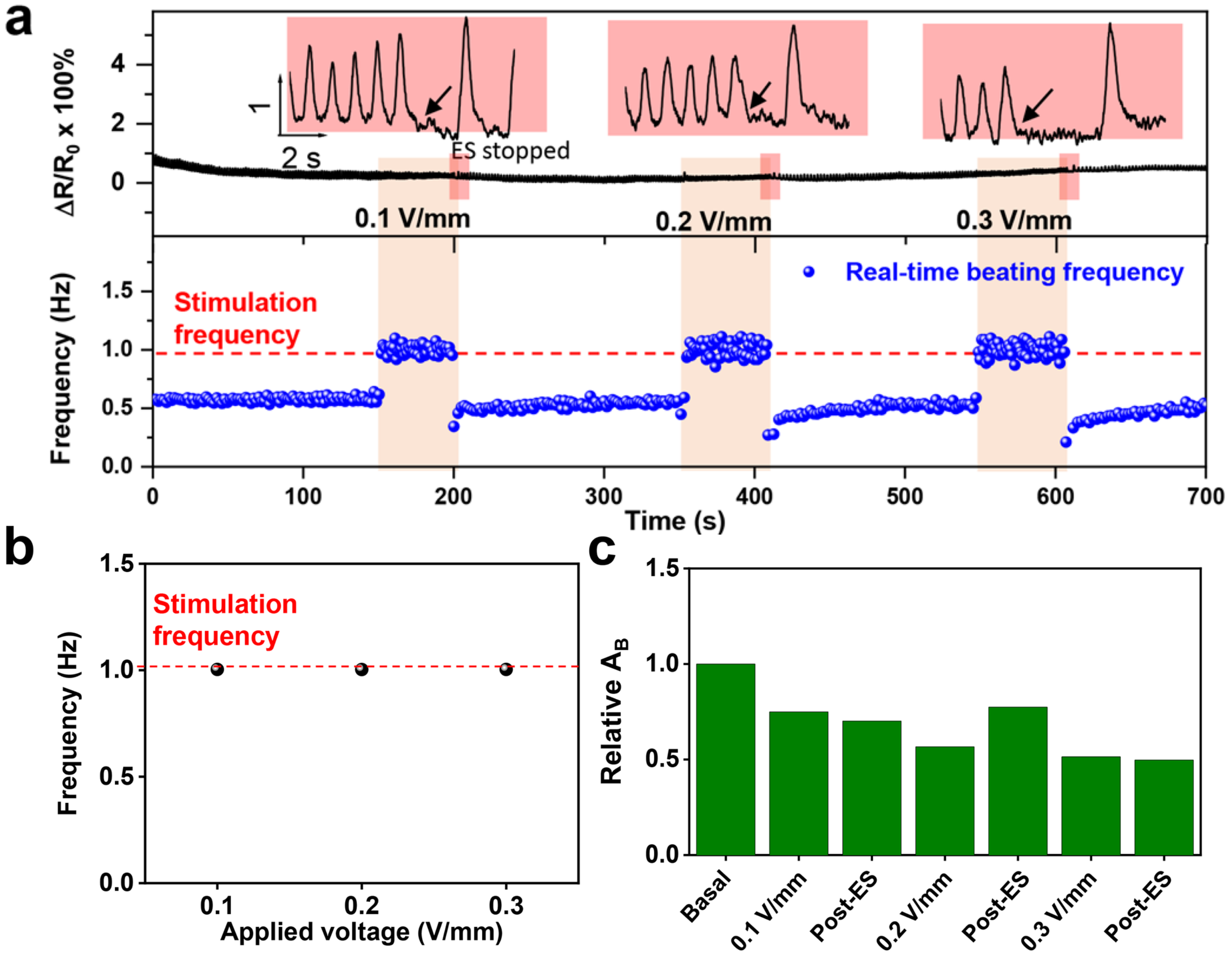

Fig. S22 **a** Top: Real-time sensor readout during the process to pace a resuscitated cardiac organoid by external electrical impulses at 1 Hz under varied field strength from 0.1 to 0.3 V mm^{-1}; Bottom: Corresponding real-time beating frequency during the process. Waveforms in red color shadings are organoid beatings during ES and right after ES. **b** The average beating frequency during ES at different applied field strength. **c** The relative beating amplitude in different periods during the stimulation process. Data are presented as mean ± SEM.

IV-6. Influence of ES frequency on pacing the resuscitated cardiac organoid

The influence of ES frequency on the resuscitated cardiac organoid is shown in Fig. S23. Although the beating pattern is synchronized with different ES frequency from 0.5-2 Hz, the resuscitated organoid showed a larger deviation in beating frequency when it was electrically stimulated (Fig. S23a).

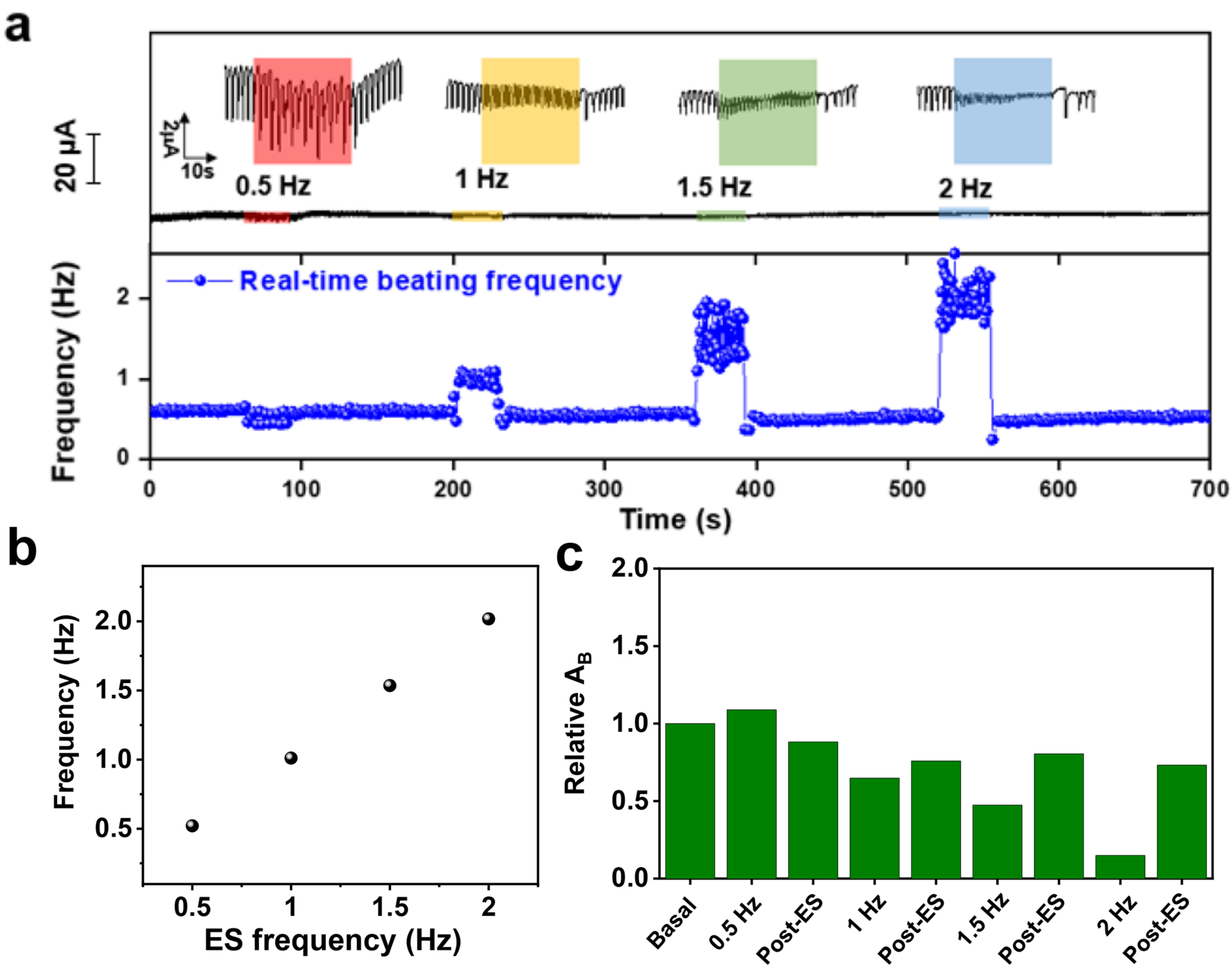

Fig. S23 a Top: Real-time sensor readout during the process to pace a resuscitated cardiac organoid by external ES at 0.5-2 Hz under an applied field strength of 0.2 V mm^{-1} for 30 seconds. Bottom: Corresponding real-time beating frequency during the process. Waveforms in the color shadings are organoid beating during electrical stimulation. **b** Average beating frequency during ES at varied frequencies. **c** Relative beating amplitude during the stimulation process. Data are mean ± SEM.

IV-7. Influence of ES duration on pacing the resuscitated cardiac organoid

The influence of ES time on the resuscitated cardiac organoid is shown in Fig. S24. The resuscitated cardiac organoid required a longer recovery duration to resume its spontaneous beating frequency post-ES when compared to a representative healthy cardiac organoid (Fig. S24b).

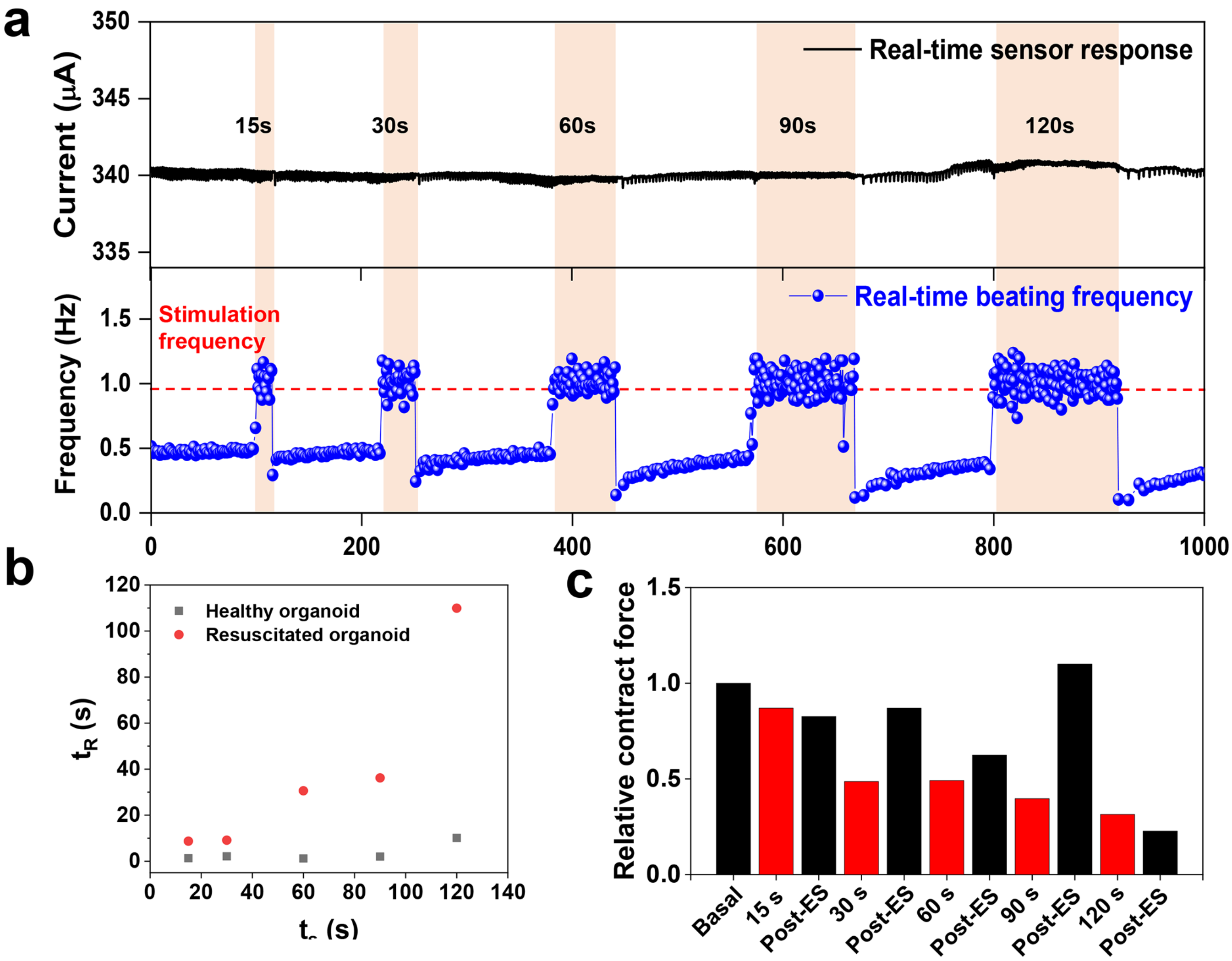

Fig. S24 **a** Top: Real-time sensor readout during the process of ES at 1 Hz for different duration (15, 30, 60, 90 and 120 seconds). Bottom: The corresponding real-time beating frequency. **b** Comparison of the recovery time of a healthy organoid and a resuscitated organoid following different ES durations. **c** The relative beating amplitude of cardiac organoid during the stimulation process. Data are presented as mean ± SEM.

IV-8. Comparison of heart rate variability (RMSSD) of a healthy organoid and a resuscitated organoid

We further compared the heart rate variability between a healthy organoid and a resuscitated organoid. The RMSSD of the resuscitated organoid was larger than that of the healthy organoid both in the non-ES and ES conditions with different ES frequency and duration (Fig. S25).

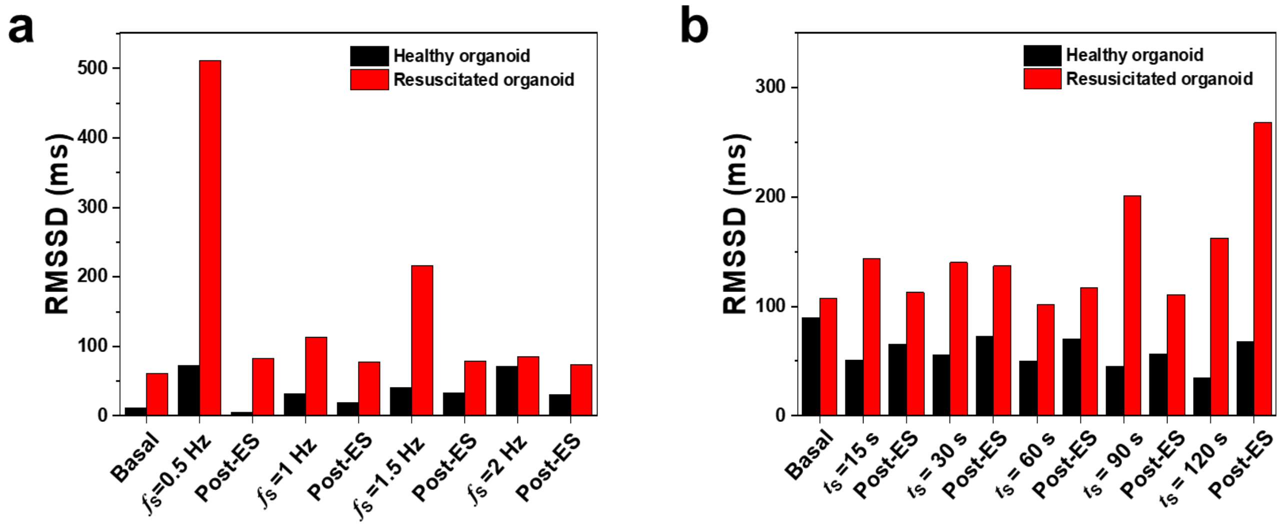

Fig. S25 Comparison of the RMSSD between a healthy organoid and a resuscitated organoid during the ES process with different ES frequencies from 0.5-2 Hz (**a**) and different ES durations from 15-120 seconds (**b**).

Section V Monitoring cardiac organoid contractility during drug dosing

To demonstrate the capability of our diaphragm sensor in detecting contractility changes of cardiac organoids in response to chronotropic and inotropic agents, cardiac organoids were treated with carbachol in a 37ºC humidified CO_2 incubator. A series of drug concentrations (from 1 nM to 10 μM) were freshly diluted from the stock solution prior to drug administration. 4 μL of carbachol was added to the 200 μL organoid media in the sensing diaphragm-integrated culture chamber. Before drug administration, the cardiac organoids were allowed to equilibrate for 1 hour following the setup of the system. Beating patterns of cardiac organoids were continuously measured throughout the process of cumulative dose-response studies (20-25 minutes incubation at each drug concentration).

The overall view of the beating frequency during the 20-minute incubation with 100 nM carbachol is demonstrated in Fig. S26, 27. The irregular beating rate was observed after carbachol solution was administrated for 5 minutes, which was continued over the next 15 minutes.

V-1. Carbachol dose-response studies

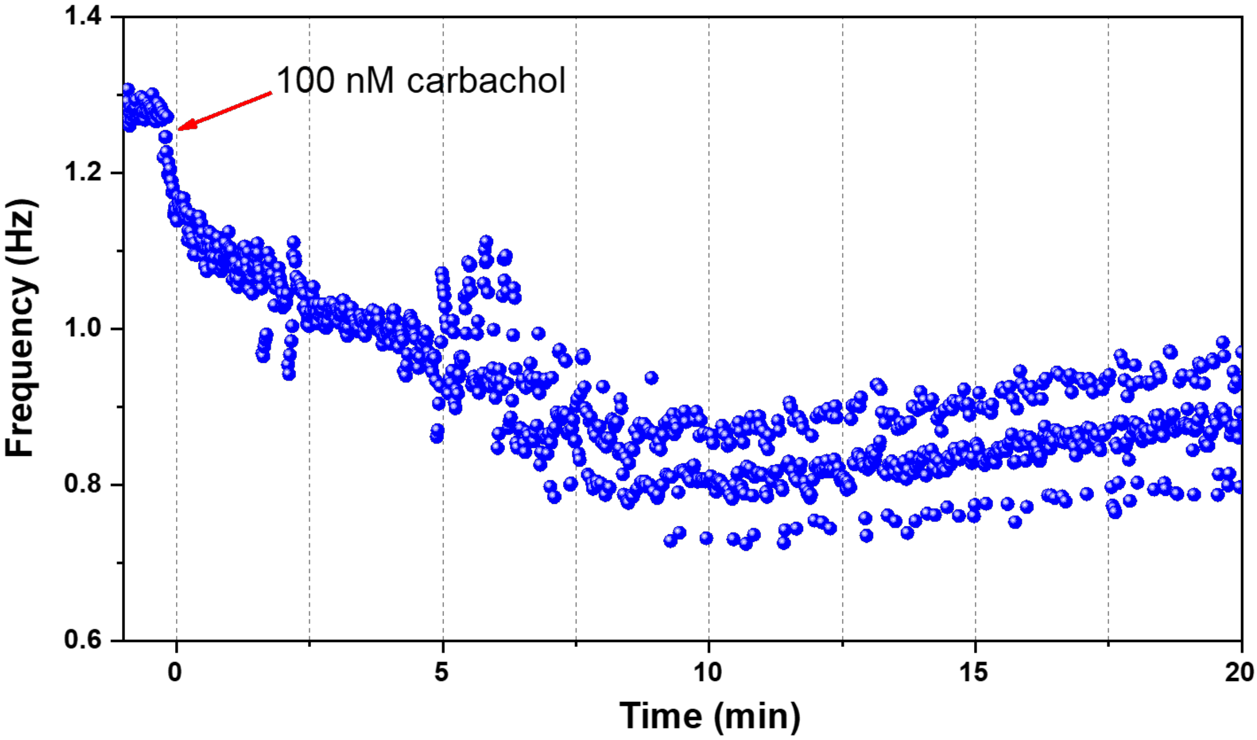

Fig. S26 Real-time beating frequency of a cardiac organoid at day 26 during a 20-minute treatment with 100 nM carbachol in an incubator at 37°C with 5% CO_2 and 95% humidity.

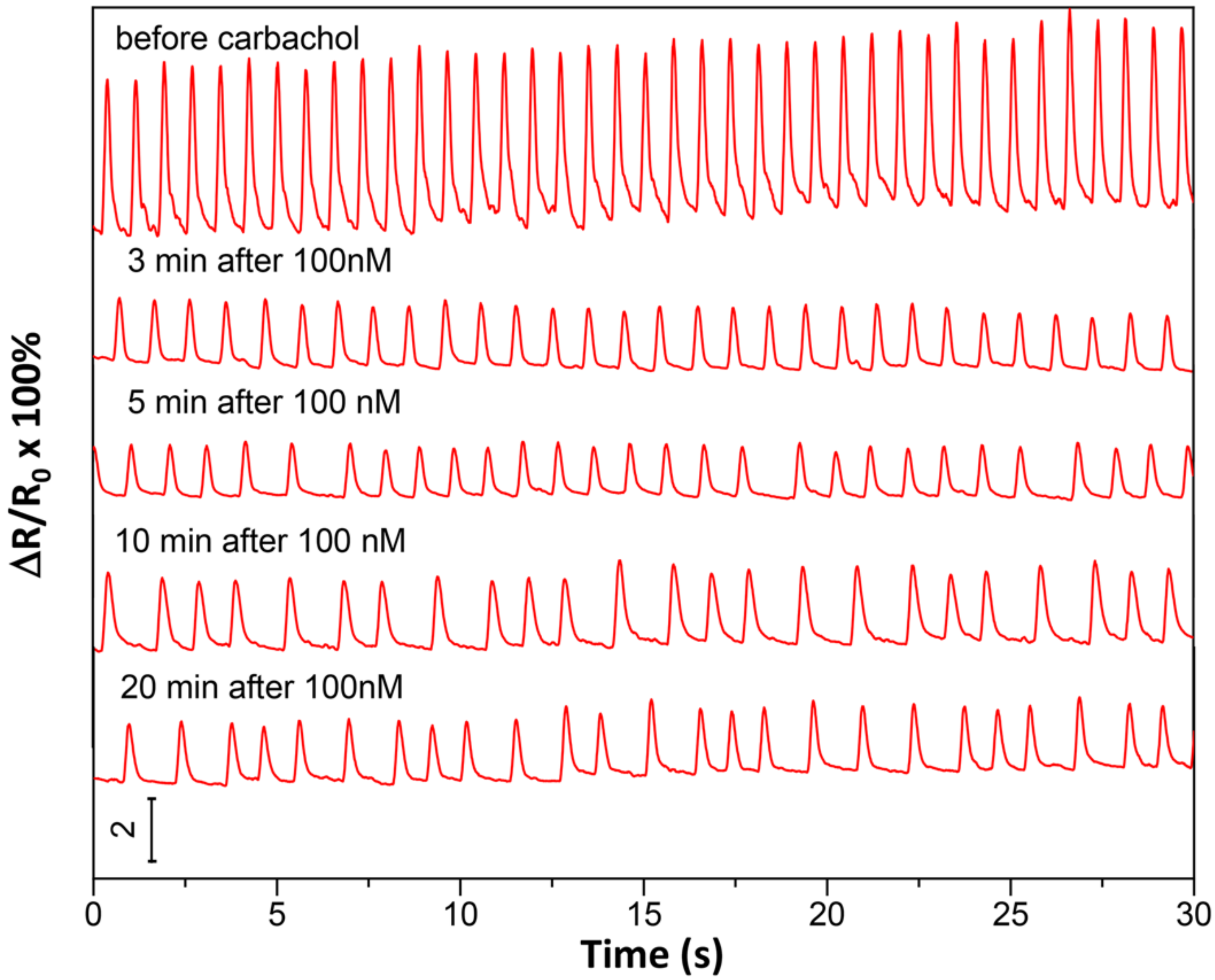

Fig. S27 Enlarged views of the changing beating patterns induced by 100 nM carbachol in a time-dependent manner.

The overall view of the beating frequency during the 20-minute incubation with 1 μM carbachol is demonstrated in Fig. S28. The irregular beating rate was observed and continued over the next 15 min after addition of 1 μM carbachol (Fig. S29). The negative chronotropic effect of carbachol reach a maximum effect at around 5 min post-treatment.

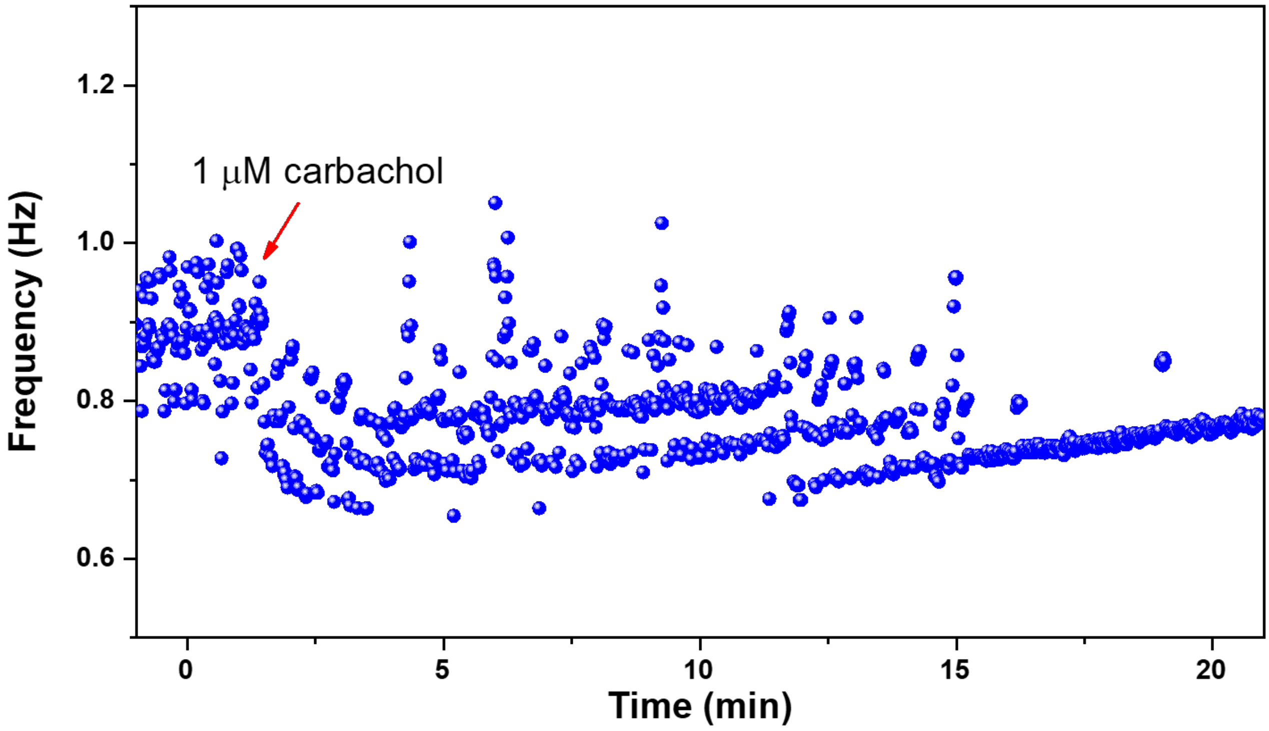

Fig. S28 Real-time beating frequency of the cardiac organoid at day 26 during a 20-minunte treatment with 1 μM carbachol in an incubator at 37°C with 5% CO_2 and 95% humidity.

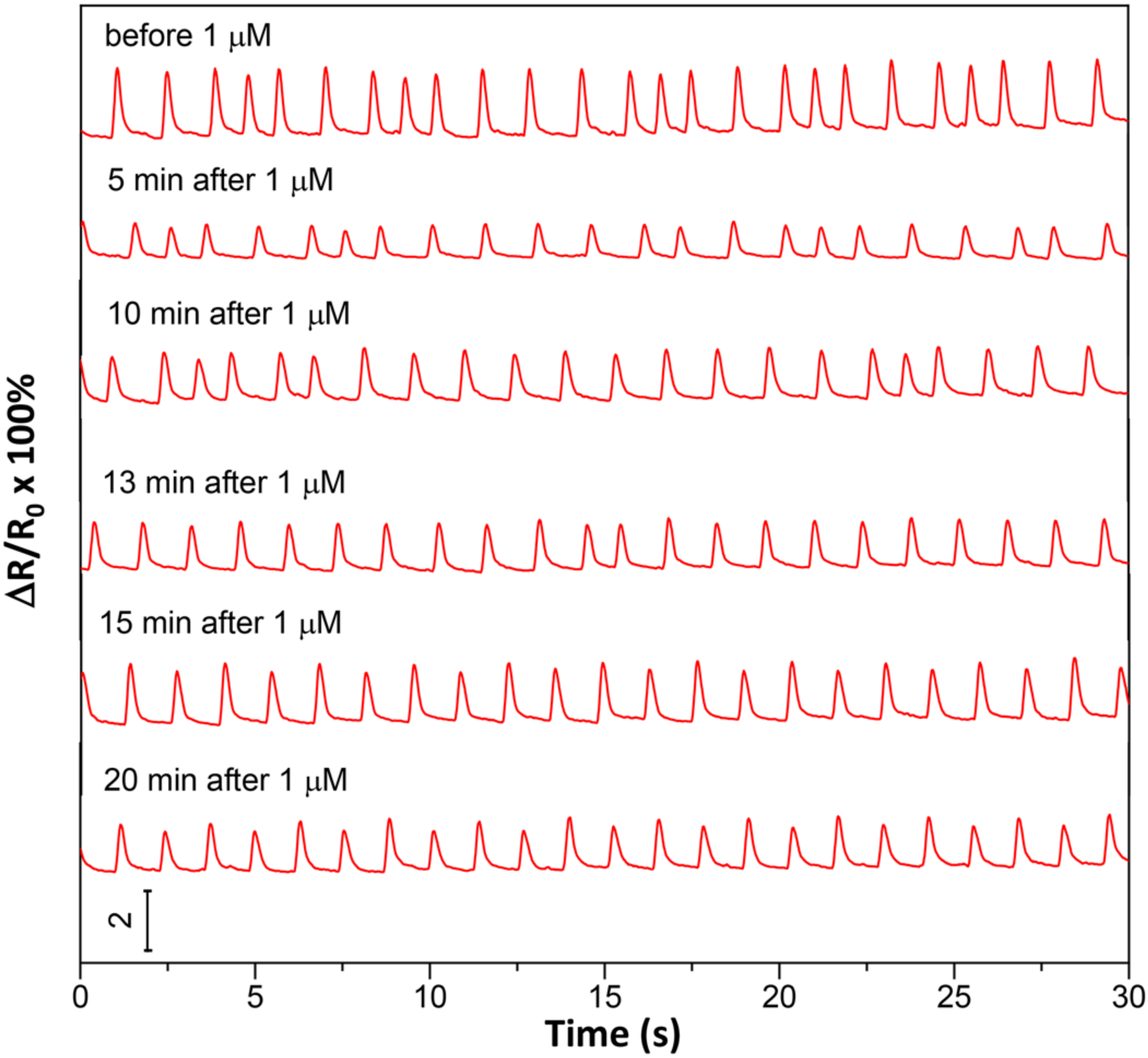

Fig. S29 Enlarged views of the changing beating patterns induced by 1 μM carbachol in a time-dependent manner.

Fig. S30 shows the beating frequency of a cardiac organoid treated with 10 µM carbachol over 23 minutes of incubation. Although the negative chronotropic effect of 10 µM carbachol was less than those induced by lower concentrations of carbachol (100 nM and 1 µM) during the early phase post-treatment, 10 µM carbachol induced severe arrhythmia after 17 min post-treatment. At this stage, the carbachol-treated cardiac organoid became very vulnerable to external mechanical disturbances and lost spontaneous beating activity following three media changes to wash out the carbachol (Fig. S31).

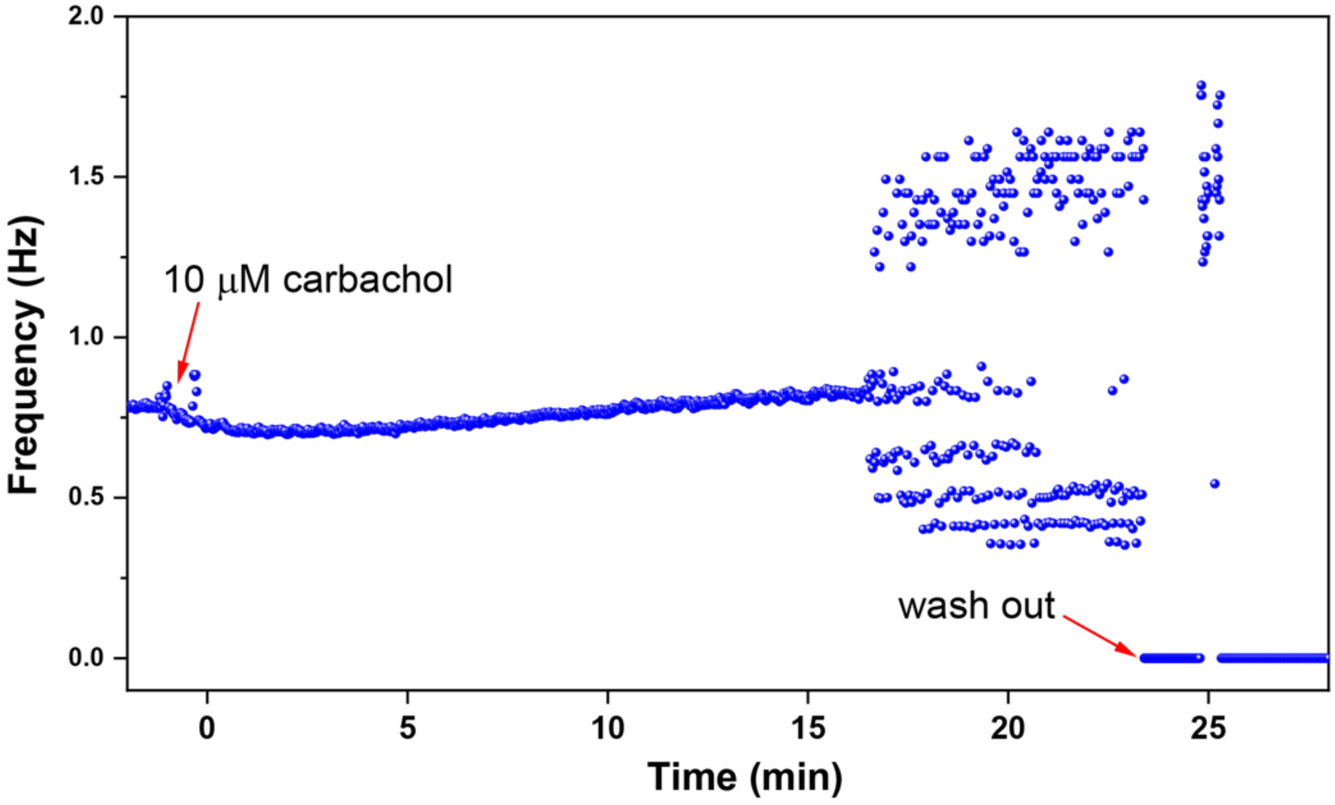

Fig. S30 Real-time beating frequency of the cardiac organoid at day 26 during a 20-minute treatment with 10 µM carbachol in an incubator at 37°C with 5% CO_2 and 95% humidity.

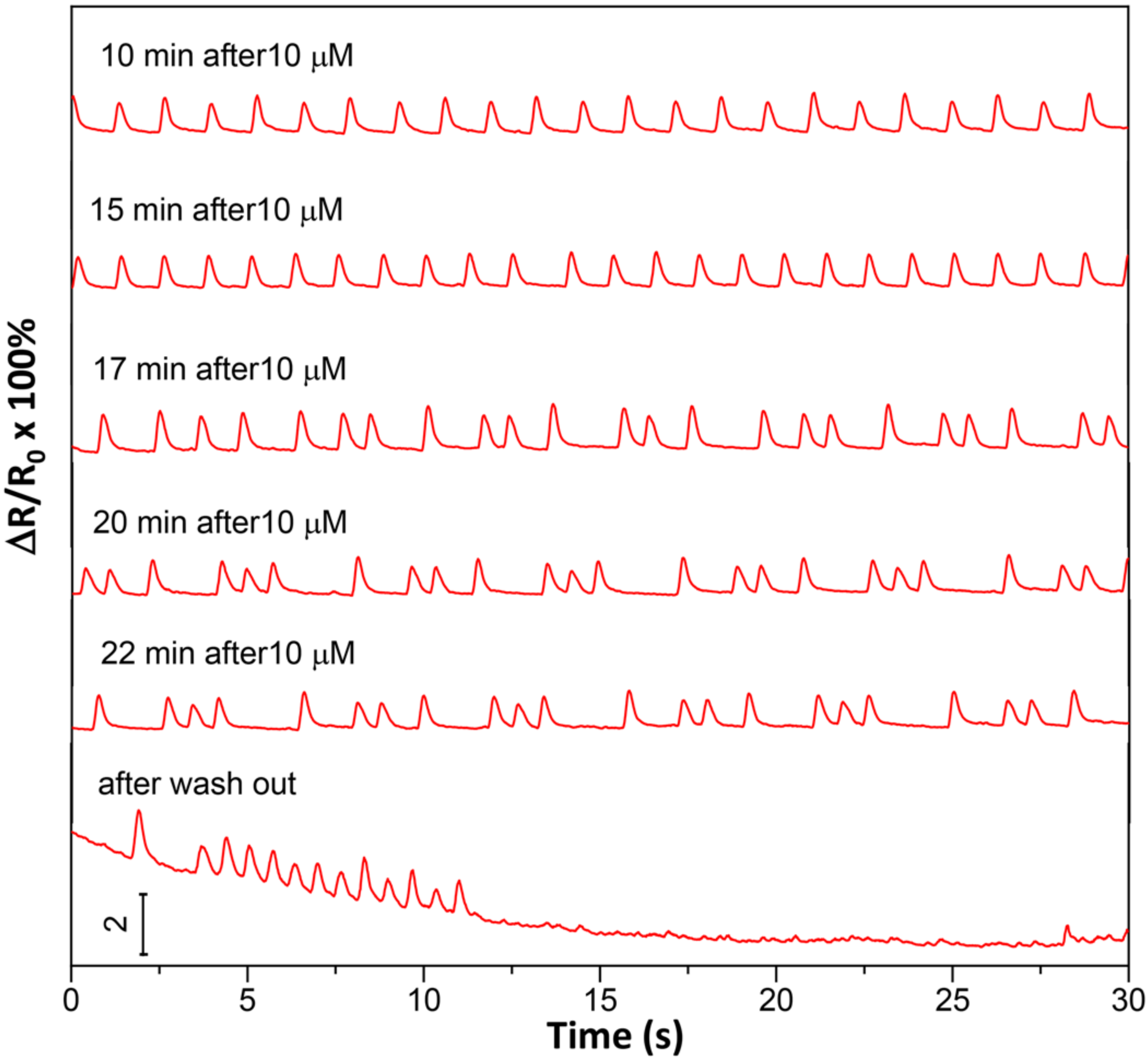

Fig. S31 Enlarged views of the changing beating patterns induced by 10 μM carbachol in a time-dependent manner.

V-2. ES on the carbachol-treated cardiac organoid

To validate the capability of our diaphragm sensor to monitor more complex contraction patterns in an incubator, we further applied ES on the carbachol-treated cardiac organoid. This organoid was successfully resuscitated and recovered its spontaneous beating with ES at 2 Hz under 0.2 V mm^{-1} for 1 minute. However, the recovered spontaneous beating rate was irregular. Hence, in order to investigate the influence of ES on this resuscitated cardiac organoid, ES at varied frequencies was further applied.

The results show that cardiac organoid could not fully capture the electrical pulses at frequencies (0.5 Hz and 1 Hz) less than its intrinsic f_B presenting non-synchronized f_B with f_S. However, the beating rate was consistent with the applied f_S, when f_S was no less than 1.5 Hz (Fig. S32 a, b). The cardiac organoid recovered its spontaneous beating pattern after removal of applied ES. However, the t_R varied depending on the applied f_S. The higher f_S, the longer t_R to recover to its basal beating (Fig. S32c). Similar to pacing the healthy organoid, a negative force-frequency relationship was also observed in this carbachol-treated cardiac organoid (Fig. S32d).

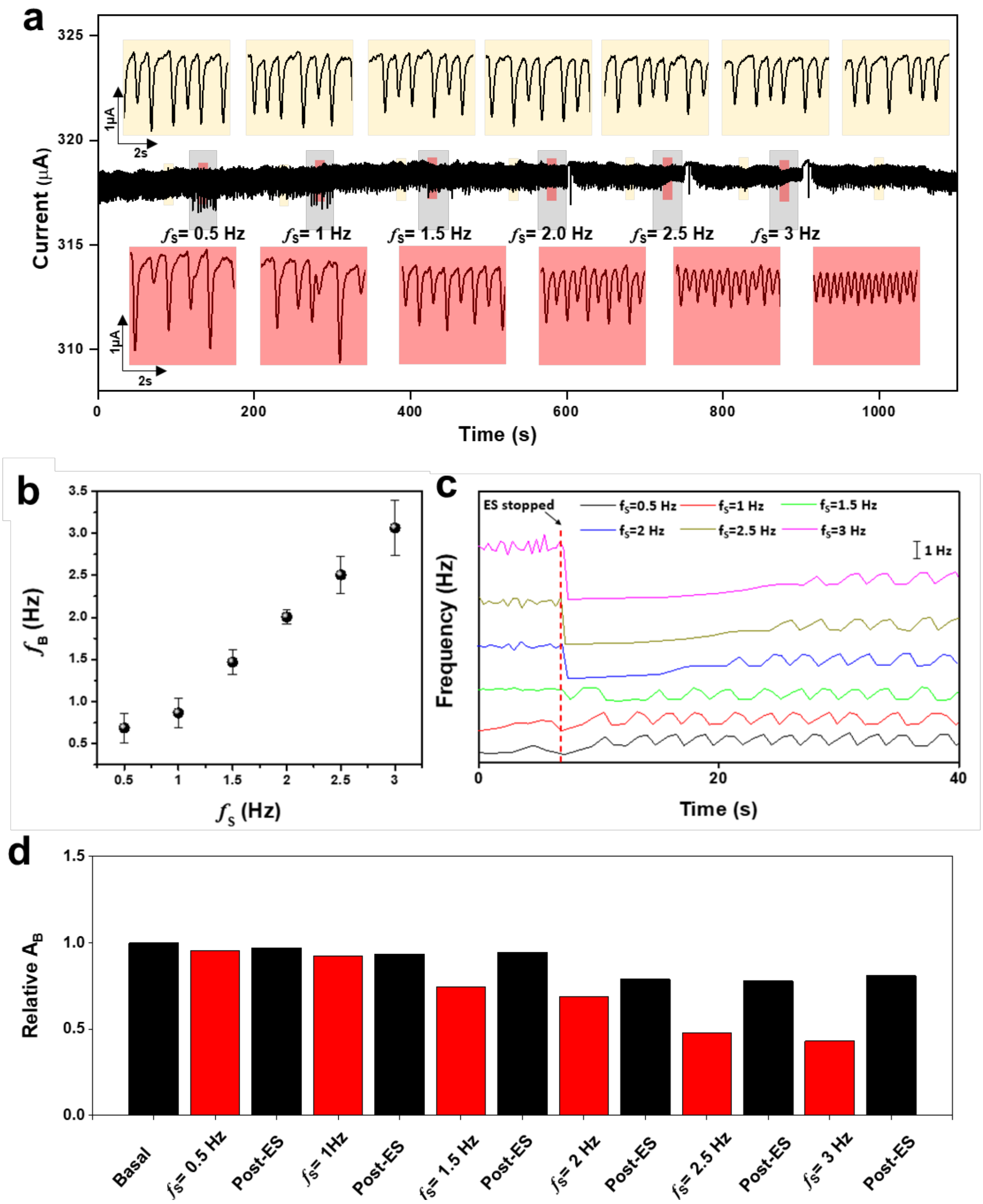

Fig. S32 **a** Relative resistance changes of the sensor in response to the carbachol-treated cardiac organoid. Waveforms shaded in yellow are from non-ES period and in red are from post-ES period. **b** The average paced beating frequency (f_B) during ES at varied frequencies (f_S: 0.5-3 Hz). **c** Time needed for the stimulated cardiac organoid to recover (t_R) upon the termination of ES at varied frequencies (f_S: 0.5-5 Hz). **d** The relative beating force of the cardiac organoid during ES (red) at varied stimulation frequencies and post-ES (green). Data are presented as mean ± SEM.

V-3. Administration of carbachol via media exchange method

We also compared the response of the organoid by using a medium exchange method for carbachol administration (Fig. S33a). The enlarged view (Fig. S33b) reflects the detailed process for removal of existing medium and addition of pre-mixed drug media. Although during the medium exchange process, mechanical disturbances were caused to the sensing diaphragm, it still worked excellently to monitor the beating patterns after drug administration. Similar to the dropwise method, the organoid displayed both the overall inhibition tendency and detailed real-time beating frequency (Fig. S33c) with the administration of carbachol with increased concentration. During the incubation with 1 μM and 10 μM carbachol, the beating rate was maintained at a relatively stable and low level. Notably, at the late stage of incubation with 10 μM carbachol, the beating rate started to noticeably fluctuate. Taken together, these results illustrate that our soft sensing platform works excellently in diverse conditions no matter how drugs are administrated (dropwise addition or medium exchange), and in different environments (ambient environment or inside a cell culture incubator).

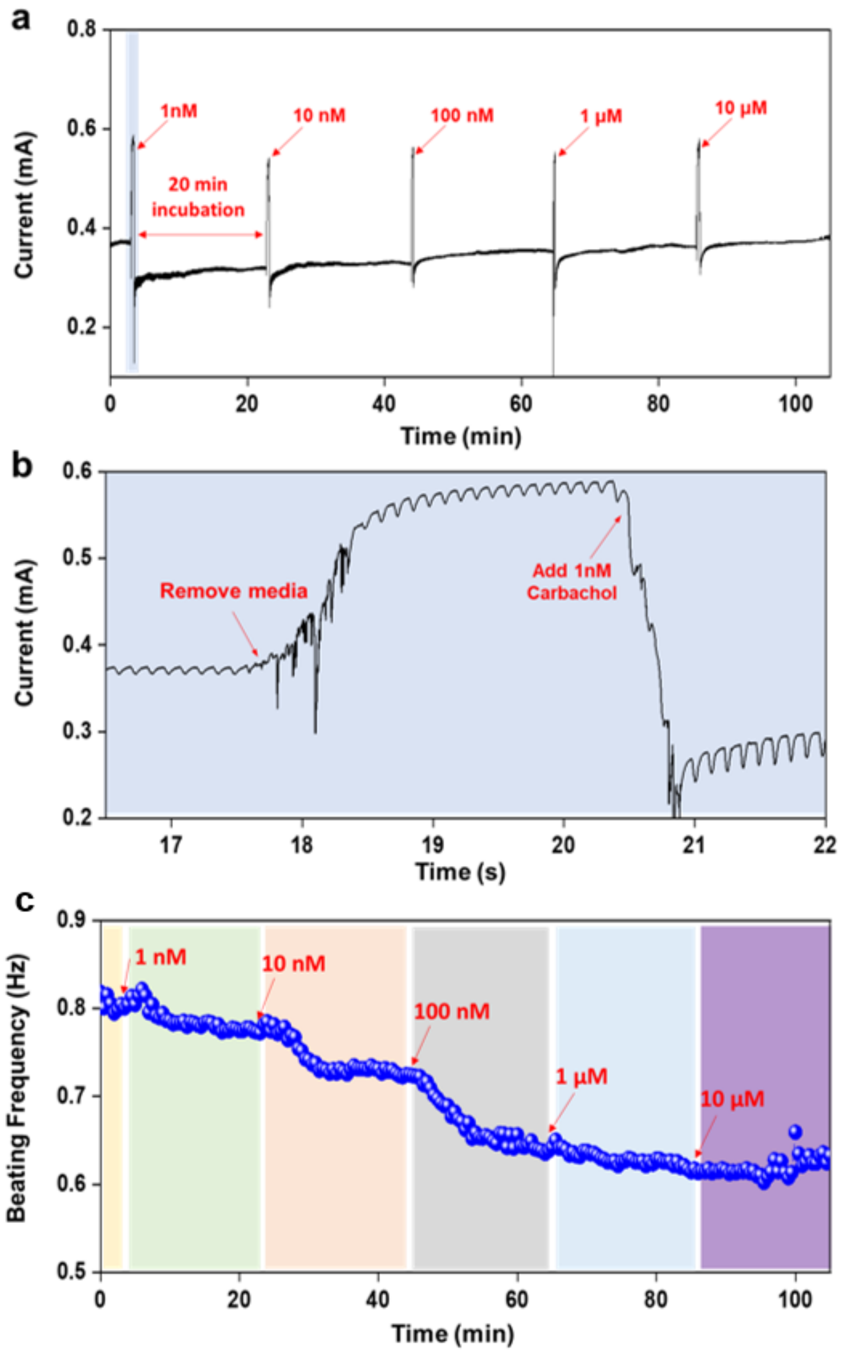

Fig. S33 Continuous assessment of the effect of increasing doses of carbachol on beating patterns of a cardiac organoid (day 15) using a medium exchange method in an ambient environment. a Continuous real-time readout of the beating events from a cardiac organoid treated with increasing doses of carbachol from 1 nM to 10 μM. **b** The enlarged view of the sensor response during the addition of 1 nM carbochol. **c** The corresponding measured real-time beating frequency during the carbachol dosing process.

Section VI FACS gating strategy for the vascularised cardiac organoids

a

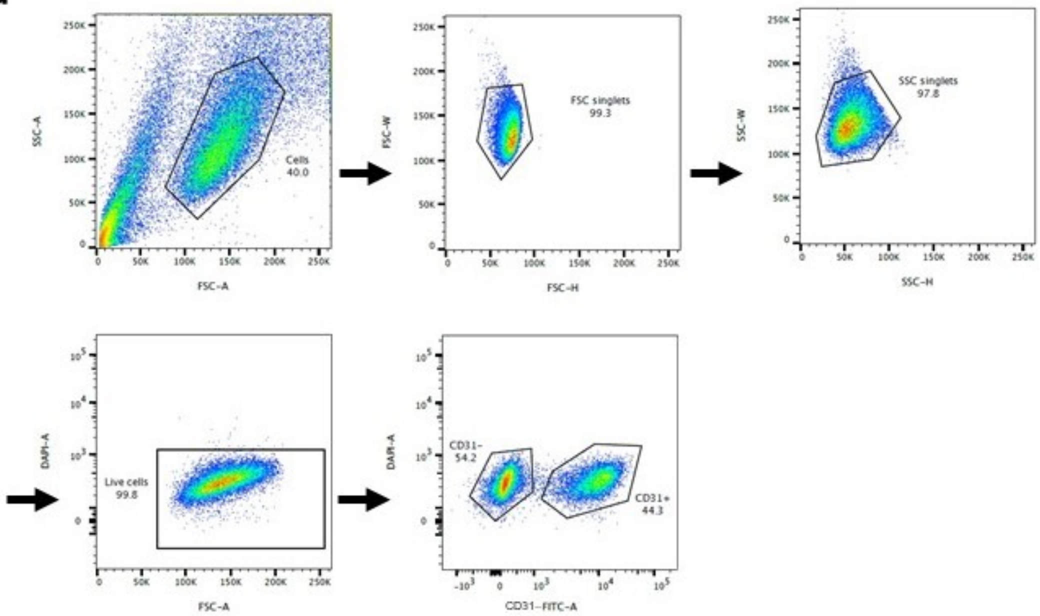

b

Name	Statistic	#Cells
Sample_Sort 1 CD31.fcs		50000
Cells	40.0	19989
FSC singlets	99.3	19853
SSC singlets	97.8	19424
Live cells	99.8	19394
CD31+	44.3	8601
CD31-	54.2	10513

Fig. S34 a FACS gating strategy for sorting CD31-positive endothelial cells for use in the vascularised cardiac organoids. Endothelial cells derived from hiPSCs were gated to remove debris, to select singlets, to select live cells, and CD31+ cells were sorted. **b** Statistics of a representative FACS experiment. Data are from three independent experiments with similar results.

Table S1. Comparison between optical microscope and force sensing diaphragm

	Optical microscope (portable microscope)	Sensor diaphragm
**Adaptive to incubator monitoring?**	Yes	Yes
**Light source required?**	Yes	No
**Sampling rate**	up to 20 Hz	>100 Hz
**Storage requirement**	>1 Mb/s	<10 Kb/s
**Contractile force?**	No	Yes

Reference

1. D. S. Kim, Y. W. Choi, A. Shanmugasundaram, Y. J. Jeong, J. Park, N. E. Oyunbaatar, E. S. Kim, M. Choi, D. W. Lee, Highly durable crack sensor integrated with silicone rubber cantilever for measuring cardiac contractility. *Nature Communications* **11**, 535 (2020).
