# Supplementary material for: A soft and ultrasensitive force sensing diaphragm for probing cardiac organoids instantaneously and wirelessly
Source: Nat Commun. 2022 Nov 25;13:7259. doi: 10.1038/s41467-022-34860-y (PMC9700778; doi:10.1038/s41467-022-34860-y)
Supplement: Supplementary file 2 — Description of additional Supplementary File [file 41467_2022_34860_MOESM2_ESM.pdf]

Descriptions of Additional Supplementary Files

Supplementary Movie 1. AFM-like engaging process for monitoring beating patterns of a cardiac organoid.

Supplementary Movie 2. Video recording of synchronized beating of an organoid.

Supplementary Movie 3. Video recording of strong beating organoids on the diaphragm sensor.

Supplementary Movie 4. Video recording of normal beating organoids on the diaphragm sensor.

Supplementary Movie 5. Video recording of weak beating organoids on the diaphragm sensor.

Supplementary Movie 6. Real-time monitoring cardiac organoid beating wirelessly via a smartphone.

Supplementary Movie 7. Real-time monitoring cardiac organoid beating pre-, during and post electrical stimulation (ES) wirelessly via a smart phone.
