# Supplementary material for: A soft and ultrasensitive force sensing diaphragm for probing cardiac organoids instantaneously and wirelessly
Source: Nat Commun. 2022 Nov 25;13:7259. doi: 10.1038/s41467-022-34860-y (PMC9700778; doi:10.1038/s41467-022-34860-y)
Supplement: Supplementary file 10 — Reporting Summary [file 41467_2022_34860_MOESM10_ESM.pdf]

nature portfolio

Corresponding author(s): Prof. Wenlong Cheng

Last updated by author(s): Oct 29, 2022

Reporting Summary

Nature Portfolio wishes to improve the reproducibility of the work that we publish. This form provides structure for consistency and transparency in reporting. For further information on Nature Portfolio policies, see our Editorial Policies and the Editorial Policy Checklist.

Statistics

For all statistical analyses, confirm that the following items are present in the figure legend, table legend, main text, or Methods section.

n/a	Confirmed	
☐	☒	The exact sample size (*n*) for each experimental group/condition, given as a discrete number and unit of measurement
☐	☒	A statement on whether measurements were taken from distinct samples or whether the same sample was measured repeatedly
☐	☒	The statistical test(s) used AND whether they are one- or two-sided<br>*Only common tests should be described solely by name; describe more complex techniques in the Methods section.*
☐	☒	A description of all covariates tested
☐	☒	A description of any assumptions or corrections, such as tests of normality and adjustment for multiple comparisons
☐	☒	A full description of the statistical parameters including central tendency (e.g. means) or other basic estimates (e.g. regression coefficient) AND variation (e.g. standard deviation) or associated estimates of uncertainty (e.g. confidence intervals)
☐	☒	For null hypothesis testing, the test statistic (e.g. *F*, *t*, *r*) with confidence intervals, effect sizes, degrees of freedom and *P* value noted<br>*Give P values as exact values whenever suitable.*
☒	☐	For Bayesian analysis, information on the choice of priors and Markov chain Monte Carlo settings
☒	☐	For hierarchical and complex designs, identification of the appropriate level for tests and full reporting of outcomes
☒	☐	Estimates of effect sizes (e.g. Cohen's *d*, Pearson's *r*), indicating how they were calculated

Our web collection on statistics for biologists contains articles on many of the points above.

Software and code

Policy information about availability of computer code

Data collection	Mechanical contractile analysis data were collected via an electrochemical workstation (PARSTAT 4000A, Princeton Applied Research).<br>Multi-electrode analysis data were collected on a MEA1060-2BC system (Multichannel Systems).<br>For FACS data collection FACS Diva Version 8.0.1 was used.
Data analysis	Video contraction data were analyzed using publicly available Image-J plugin MUSCLEMOTION v1.0 published in (Sala, L., et al., Circ Res, 2018. 122(3):p.e5-e16.<br>For data processing and statistical analysis, GraphPad Prism 9.3.1 and OriginPro 20201b softwares.<br>For FACS data analysis FLowJo v10.8 was used.

For manuscripts utilizing custom algorithms or software that are central to the research but not yet described in published literature, software must be made available to editors and reviewers. We strongly encourage code deposition in a community repository (e.g. GitHub). See the Nature Portfolio guidelines for submitting code & software for further information.

Data

Policy information about availability of data

All manuscripts must include a data availability statement. This statement should provide the following information, where applicable:

- Accession codes, unique identifiers, or web links for publicly available datasets
- A description of any restrictions on data availability
- For clinical datasets or third party data, please ensure that the statement adheres to our policy

All relevant data supporting the findings of this study are available within this article and in the Supplementary Information files. All raw and processed data are available from the corresponding author upon reasonable request. Source data are provided with this paper.

Human research participants

Policy information about studies involving human research participants and Sex and Gender in Research.

Reporting on sex and gender	No human participants were used in this study
Population characteristics	No human participants were used in this study
Recruitment	No human participants were used in this study
Ethics oversight	No human participants were used in this study

Note that full information on the approval of the study protocol must also be provided in the manuscript.

Field-specific reporting

Please select the one below that is the best fit for your research. If you are not sure, read the appropriate sections before making your selection.

- [x] Life sciences
- [ ] Behavioural & social sciences
- [ ] Ecological, evolutionary & environmental sciences

For a reference copy of the document with all sections, see nature.com/documents/nr-reporting-summary-flat.pdf

Life sciences study design

All studies must disclose on these points even when the disclosure is negative.

Sample size	Sample size was predetermined with a power calculation based on standard deviation of 10% observed in pilot experiments. A sample size of three experimental replicates was estimated to provide >80% power to detect at least a 22% change with ANOVA (SD=10%, type I error set at α=0.05 and type 2 error set at β=0.2).
Data exclusions	No data were excluded from the analysis.
Replication	Except the figures in the latest version of supplementary information files S 12h-i, 20c, 22c, 23c, 24c, 32d in which only one organoid was used, all the remaining experiments on organoid beating monitoring and sensor characterization were repeated three independent times to confirm the repeatability of the findings. Data presented in this study is representative for the different repeated measurement that combined in one final figure from three independent experiments with similar results.
Randomization	Biological tissue samples were randomly allocated to treatment groups.
Blinding	Blinding was not performed in this study.

Reporting for specific materials, systems and methods

We require information from authors about some types of materials, experimental systems and methods used in many studies. Here, indicate whether each material, system or method listed is relevant to your study. If you are not sure if a list item applies to your research, read the appropriate section before selecting a response.

Materials & experimental systems

n/a	Involved in the study
	☒ Antibodies
	☒ Eukaryotic cell lines
☒	Palaeontology and archaeology
☒	Animals and other organisms
☒	Clinical data
☒	Dual use research of concern

Methods

n/a	Involved in the study
☒	ChIP-seq
	☒ Flow cytometry
☒	MRI-based neuroimaging

Antibodies

Antibodies used

Primary-conjugated antibody: FITC Mouse Anti-Human CD31 (Endothelial), BD Pharmingen, #555445, clone WM59, lot#8212882
Primary Antibody: Troponin T (Cardiac), Abcam, ab45932, polyclonal, lot#GR3394585-1
Primary Antibody: CD31 (Endothelial Cell), DAKO, M0823, clone JC70A, lot#00093760
Secondary antibody: Alexa Fluor-488-conjugated goat-anti-mouse, Invitrogen, A11001, Lot#2247988
Secondary antibody: Alexa Fluor-594-conjugated goat-anti-rabbit, Invitrogen, A11005, Lot#2043369

Validation

Primary-conjugated antibody ITC Mouse Anti-Human CD31 (Endothelial) has been extensively validated in the literature as described on the supplier's product information sheet (https://www.bdbiosciences.com/content/bdb/paths/generate-tds-document.us.555445.pdf). The details of this validation are as follows: The WM59 monoclonal antibody specifically binds to platelet endothelial cell adhesion molecule-1, (PECAM-1, PECAM1), which is also known as GPIIA', or EndoCAM. CD31 is a 130 kDa type I transmembrane glycoprotein that belongs to the Ig gene superfamily. CD31 has wide tissue distribution and is expressed on platelets, monocytes, granulocytes, NK cells, T cell subsets, and in high amounts on endothelial cells. CD31 functions as a vascular endothelial cell adhesion molecule and is involved in the transendothelial migration of leucocytes in inflammatory responses. It might be involved in thrombosis, angiogenesis, and wound healing. The WM59 appears to recognize an epitope proximal to extracellular domain 2 of CD31. We have also published using this antibody before: Kong, Anne M et al. “Bio-engineering a tissue flap utilizing a porous scaffold incorporating a human induced pluripotent stem cell-derived endothelial cell capillary network connected to a vascular pedicle.” Acta biomaterialia vol. 94 (2019): 281-294. doi:10.1016/j.actbio.2019.05.067

Primary Antibody: Troponin T (Cardiac), has been extensively validated in human heart samples as shown on the product website (https://www.abcam.com/Cardiac-Troponin-T-antibody-ab45932.html?gclsrc=aw.ds|aw.ds&=v&gclid=Cj0KCQjwidSWBhDdARIsAIoTVb1yIDxoGimjE1jd1wDrFnaTsOb29pcynT8Q6kSe2VN0eX-rAJih_NkaAivDEALw_wcB), and published over 71 times. We have also published using this antibody before: Kompa, Andrew R et al. “Sustained subcutaneous delivery of secretome of human cardiac stem cells promotes cardiac repair following myocardial infarction.” Cardiovascular research vol. 117,3 (2021): 918-929. doi:10.1093/cvr/cvaa088

Primary Antibody: CD31 (Endothelial Cell), has been extensively validated in multiple human tissue samples as shown on the product website (https://www.agilent.com/en/product/immunohistochemistry/antibodies-controls/primary-antibodies/cd31-endothelial-cell-%28concentrate%29-76539#specifications). The antibody was clustered as anti-CD31 at the Fifth International Workshop and Conference on Human Leucocyte Differentiation Antigens (4). The epitope recognized was found to be within the extracellular domain 1 (3). In Western blotting of membrane preparations from a spleen rich in the antigen or from normal platelets, the antibody labels bands of respectively 100 kDa and 130 kDa, the latter corresponding to classic CD31. The smaller band of 100 kDa observed with the splenic preparation may be due to proteolytic breakdown or to variations in glycosylation (1). See package insert for reference(s). We have also published using this antibody before: Kong, Anne M et al. “Bio-engineering a tissue flap utilizing a porous scaffold incorporating a human induced pluripotent stem cell-derived endothelial cell capillary network connected to a vascular pedicle.” Acta biomaterialia vol. 94 (2019): 281-294. doi:10.1016/j.actbio.2019.05.067

Eukaryotic cell lines

Policy information about cell lines and Sex and Gender in Research

Cell line source(s)

Human induced pluripotent stem cell-Foreskin-2 cell line (male).

Authentication

The cell line used in this study has been extensively authenticated since its derivation in 2007 by James Thompson. This includes normal karyotypes, express telomerase activity, express cell surface markers and genes that characterize human ES cells, and maintain the developmental potential to differentiate into advanced derivatives of all three primary germ layers. (Yu, Junying et al. “Induced pluripotent stem cell lines derived from human somatic cells.” Science (New York, N.Y.) vol. 318,5858 (2007): 1917-20. doi:10.1126/science.1151526)

Mycoplasma contamination

All cell lines tested negative for mycoplasma

Commonly misidentified lines
(See ICLAC register)

No cell lines used in this study are commonly misidentified.

Flow Cytometry

Plots

Confirm that:

- [x] The axis labels state the marker and fluorochrome used (e.g. CD4-FITC).
- [x] The axis scales are clearly visible. Include numbers along axes only for bottom left plot of group (a 'group' is an analysis of identical markers).
- [x] All plots are contour plots with outliers or pseudocolor plots.
- [x] A numerical value for number of cells or percentage (with statistics) is provided.

Methodology

Sample preparation	As described in the Methods section, hiPSC-derived endothelial cells were trypsinised using Tryple Select (Gibco) to generate single cells, resuspended in FACS buffer (0.5 % (w/v) BSA, 2 mM EDTA in DPBS) and incubated with conjugated FITC Mouse Anti-Human CD31 (Catalogue #555445, BD Pharmingen) for 30 mins at room temperature in the dark. Stained cells were resuspended in FACS buffer and passed through a 40 um mesh. An unstained cell sample was used as a negative control for gating.
Instrument	BD FACS Aria III
Software	FACS Diva Version 8.0.1
Cell population abundance	CD31-positive cells constituted 44.3% of the live single cell events sorted. Positive/negative events were determined using an unstained cell population to determine gates.
Gating strategy	1) FSC-A vs SSC-A to remove debris, 2) FSC-H vs FSC-W and 3) SSC-H vs SSC-W to select single cells, 4) DAPI-A vs FSC-A to select live cells, and 5) CD31-FITC-A vs DAPI-A to select CD31-positive cells using an unstained cell sample to identify negative staining.

- [x] Tick this box to confirm that a figure exemplifying the gating strategy is provided in the Supplementary Information.
